# Synthesis of Novel Tetra-Substituted Pyrazole Derivatives Using Microwave Irradiation and Their Anti-Leukemic Activity Against Jurkat Cells

**DOI:** 10.3390/molecules30132880

**Published:** 2025-07-07

**Authors:** Felipe P. Machado, Maria Clara Campos, Juliana Echevarria-Lima, Diego P. Sangi, Carlos Serpa, Otávio Augusto Chaves, Aurea Echevarria

**Affiliations:** 1 Department of Organic Chemistry, Institute of Chemistry, Federal Rural University of Rio de Janeiro, Seropédica 23897-000, RJ, Brazil; felipepires@ufrrj.br; 2 Paulo de Góes Institute of Microbiology, Federal University of Rio de Janeiro, Rio de Janeiro 21941-902, RJ, Brazil; mariaclara.scampos@gmail.com (M.C.C.); juechevarria@micro.ufrj.br (J.E.-L.); 3 Institute of Exact Sciences, Fluminense Federal University, Volta Redonda 27213-145, RJ, Brazil; dpsangi@id.uff.br; 4 Department of Chemistry, Coimbra Chemistry Centre—Institute of Molecular Science, University of Coimbra, Rua Larga, 3004-535 Coimbra, Portugal; serpasoa@uc.pt; 5 Laboratory of Immunopharmacology, Centro de Pesquisa, Inovação e Vigilância em COVID-19 e Emergências Sanitárias, Oswaldo Cruz Institute, Oswaldo Cruz Foundation, Rio de Janeiro 21040-361, RJ, Brazil

**Keywords:** pyrazole, ketene, dithioacetals, microwave-assisted, in-silico predictions, leukemia, Jurkat cells

## Abstract

Three previously synthesized ketene dithioacetals were used as intermediates to obtain four nucleophiles to synthesize ten tetra-substituted pyrazoles (**11**–**20**). This was achieved through microwave irradiation in ethanol as the solvent, yielding superb results ranging from 68.4% to 90.1%, in agreement with some of the principles of green chemistry. The proposed structures were determined using various spectroscopic techniques, including infrared spectroscopy and hydrogen and carbon-13 nuclear magnetic resonance. Furthermore, the compounds underwent in-silico evaluations using CLC-Pred and AdmetSAR software to predict the absorption, distribution, metabolism, excretion, and toxicity (ADMET) properties. This was combined with molecular docking calculations for four main cancer-related targets for pyrazole core, to facilitate screening for subsequent biological assessments. Based on the data generated from these analyses, it was identified two pyrazoles (**11** and **18**) likely to exhibit anti-tumor activity, while also demonstrating low toxicity levels. Upon selection, these two pyrazoles were subjected to toxicity assessments using the *Artemia salina* method and evaluated for their effects on the viability of Jurkat cancer cells with a potency of 45.05 and 14.85 µM to **11** and **18**, respectively, and with a potency of above 100 µM for the non-carcinogenic cells HEK 293. Overall, the findings from these studies indicate pyrazole derivatives as potential anti-tumor candidates.

## 1. Introduction

Pyrazoles are exceptionally versatile five-membered aromatic heterocycles known for their significant therapeutic properties [1,2,3,4]. Their fundamental structure features two adjacent nitrogen atoms, which impart unique characteristics enabling the pyrazole core to act as either a hydrogen donor or an acceptor, depending on the reaction environment, polarity, and aqueous solubility [5]. Additionally, pyrazoles exhibit important structural features, including aromaticity and prototropic tautomerism. The amphoteric nature of the pyrazole core presents a significant advantage from a synthetic perspective as it allows for the easy introduction of various functional groups [6]. For these reasons, pyrazole derivatives have been widely explored in drug development for treating multiple diseases, having analgesic, antibacterial, antihyperglycemic, anti-inflammatory, and anti-tumor properties [7]. Some clinically used drugs contain a pyrazole ring, e.g., the nonsteroidal anti-inflammatory celecoxib [8], analgesic difenamizole, anti-obesity rimonabant, antipsychotic 3-cyano-*N*-(1,3-diphenyl-1 *H*-pyrazol-5-yl)benzamide (CDPPB) [9], and the anabolic steroid stanozolol, which is used to manage hereditary angioedema [10]. Recently, the pyrazole edaravone was explored as a free radical scavenger to slow the progression of amyotrophic lateral sclerosis [11], reinforcing the biological application of this heterocyclic core. Additionally, different reports indicated that pyrazole derivatives possess good inhibitory activities against various targets in cancer cells [12]. They were also considered an important scaffold for the design of anti-tumor drugs, such as the commercial drugs axitinib and entrectinib, which are used as kinase inhibitors in the treatment of advanced renal cell carcinoma and for treating metastatic non-small cell lung cancer, respectively [13].

**Scheme 1:**
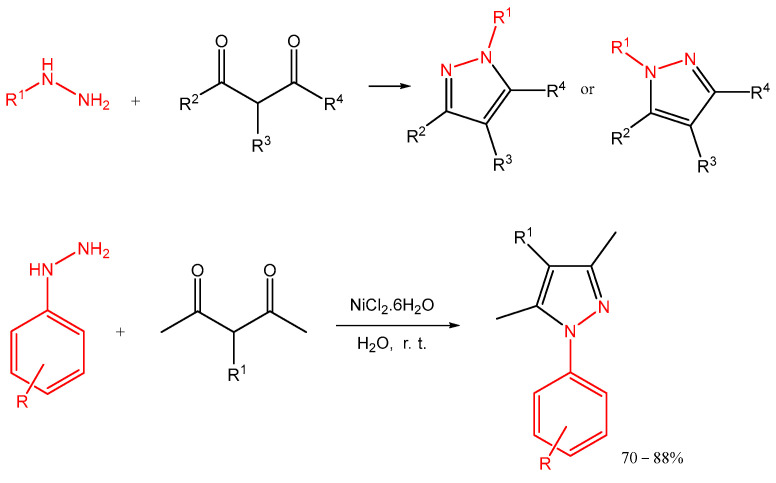
Synthesis of pyrazoles catalyzed by nickel chloride using the Knorr method [15].

The pyrazole core present in many bioactive compounds can be synthesized through various versatile methods that enable the incorporation of different functional groups. The Paal–Knorr synthesis is one of the classic approaches for obtaining the pyrazole nucleus, involving a cyclo-condensation reaction between 1,3-dicarbonyl compounds and hydrazines [14]. However, its primary limitation lies in the potential formation of a mixture of isomeric products when the substituents R^2^ and R^4^ differ. Recently, Konwar and collaborators [15], following an investigative study on various solvents and catalysts, developed an improved protocol. They applied this method to the reactions between 1,3-diketones and hydrazines, yielding a series of pyrazole derivatives with good to excellent results (Scheme 1).

**Scheme 2:**
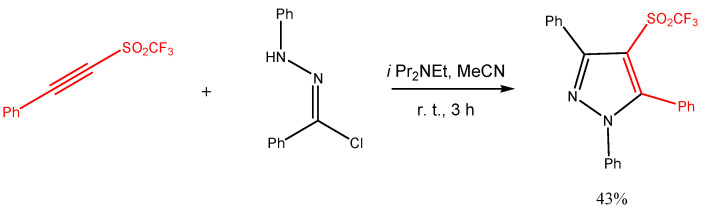
Synthesis of 1,3,5-triphenyl-4-trifluorosulfonylpyrazole [17].

The Pechmann synthesis is a well-established method for synthesizing the pyrazole nucleus through 1,3-dipolar cycloaddition involving alkynes and diazo compounds. However, this approach has the drawback of generating by-products when the alkyne utilized possesses different substituents attached to the sp carbons [16]. Several pyrazole-containing molecules have been synthesized from alkynes and diazo compounds using alternative methodologies derived from the Pechmann method. For instance, Kawai and collaborators [17] employed (trifluoromethyl)-sulfonyl-ethynylbenzene and (*E*)-*N*-phenylbenzene-carbohydrazonoyl chloride as starting materials in acetonitrile, successfully producing a poly-substituted pyrazole nucleus with a yield of 43% (Scheme 2).

**Scheme 3:**
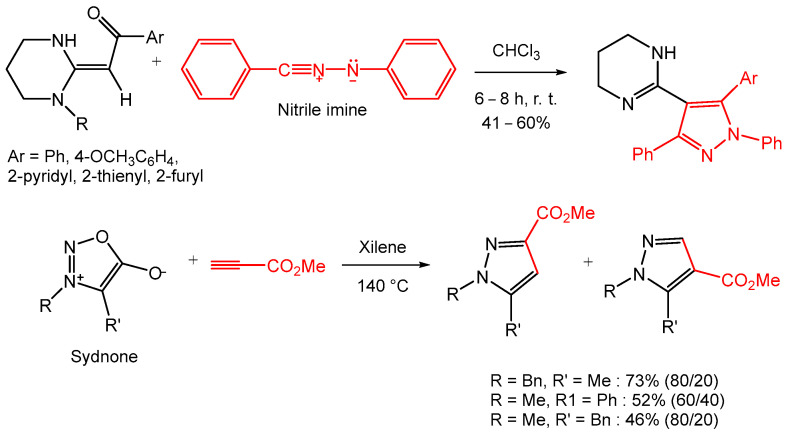
Synthesis of pyrazoles using nitrile imine [18] and sydnones [19], respectively.

The 1,3-dipolar cycloadditions are among the most important methods for synthesizing various heterocyclic rings, including pyrazoles, in a concerted manner. Nitrile imines and sydnones can act as dipolarophiles, replacing alkynes or alkenes in these reactions [18,19]. Scheme 3 provides examples of these processes.

**Scheme 4:**
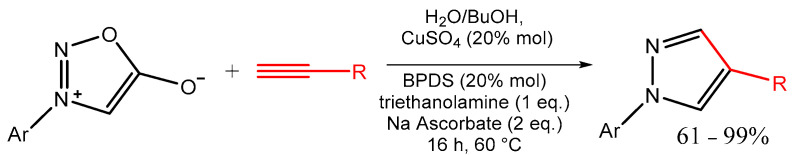
Cycloaddition reaction between sydnone and alkyne with Cu(II) as a catalyst in aqueous conditions [20].

The synthesis of pyrazoles from sydnones via thermal cycloadditions with alkynes often suffers from a lack of regioselectivity. To overcome this challenge, the reaction can be performed with catalytic amounts of Cu(II) under mild conditions, resulting in the formation of 1,4-disubstituted pyrazoles with improved yields and enhanced chemoselectivity. An example of this process is illustrated in Scheme 4 [20].

The in-situ generation of dipolarophiles has been documented. For example, Szilágyi and collaborators [21] began with ethyl glycinate hydrochloride to produce ethyl diazoacetate, which was subsequently used to prepare ethyl 5-acetyl-1*H*-pyrazole-3-carboxylate, an essential intermediate in the synthesis of darolutamide. It is important to highlight that alternative methodologies for preparing the pyrazole ring include sequential condensation, Michael addition, and intramolecular cyclization reactions [22]. Furthermore, α-sulfenylation of 1,3-dicarbonyl compounds can be achieved using potassium iodide (KI) and *tert*-butyl hydroperoxide (TBHP) with toluene as solvent under reflux [23], which is not a green approach.

Based on the biological relevance of pyrazole core and the importance of the development of easy and green methods to synthesize pyrazole derivatives, this study reports the synthesis of ten tetra-substituted pyrazoles (compounds **11**–**20**) using three nitrile intermediates, i.e., methyl 3,3-*bis*(methylsulfanyl)-2-cyanoacrylate (**4**), 2-benzoyl-3,3-*bis*(methylsulfanyl)-acrylonitrile (**5**), and 2-((*bis*-methylsulfanyl)methylene-malononitrile (**6**), under microwave irradiation in the presence of polar solvent. Furthermore, the obtained tetra-substituted pyrazoles were predicted in silico in terms of absorption, distribution, metabolism, excretion, and toxicity (ADMET) with AdmetSAR and CLC-Pred software. This was combined with molecular docking calculations for four main cancer-related targets associated with the pyrazole core [24,25,26,27]. Finally, in vitro assays on toxicity through the *Artemia salina Leach* survival and the cytotoxic to human T lymphocyte cell (Jurkat leukemia cells) and Human Embryonic Kidney (HEK) 293 cells (non-carcinogenic cells) were carried out on the best-predicted compounds to validate the in-silico data.

## 2. Results and Discussion

### 2.1. Synthesis

**Scheme 5:**
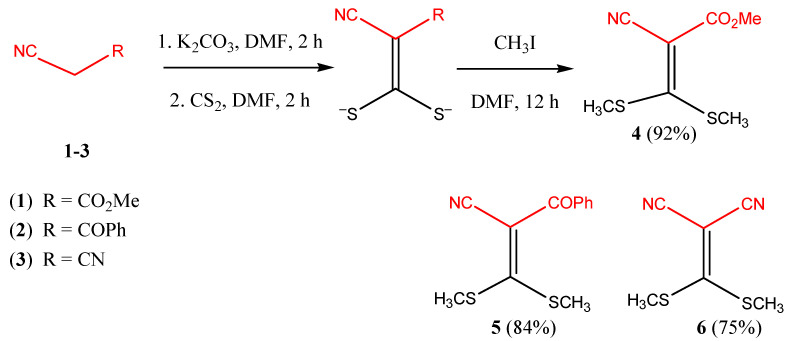
Synthesis of nitrile intermediates **4**–**6**.

Nitrile precursors (**4**–**6**) were synthesized through a three-step reaction process without isolating the intermediates. The study explored the acidic nature of the hydrogen alpha to the nitrile group in the presence of carbonate, noting a color change from whitish to reddish orange. After deprotonation, carbon disulfide was added to the reaction mixture. The final step involved an alkylation reaction, where methyl iodide was used to alkylate the sulfur atoms. Following the reaction, extraction was performed using dichloromethane, and the organic phase was washed with distilled water. The organic layer was then dried under vacuum, yielding amorphous solids with colors ranging from yellow to ocher in good yields, as illustrated in Scheme 5. These intermediates have been documented in the literature and were confirmed by their melting points and infrared spectra [28,29,30,31].

**Scheme 6:**
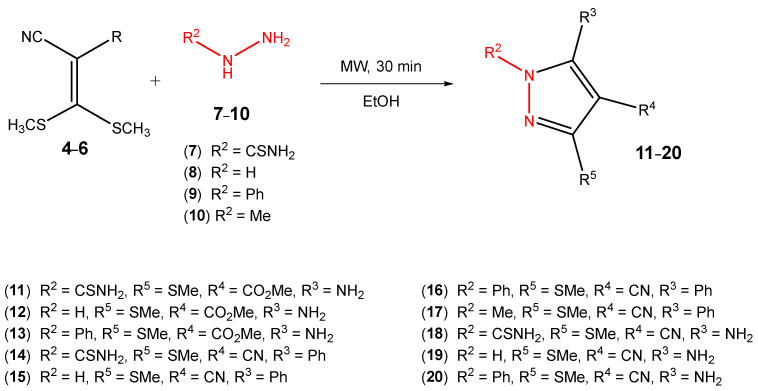
Preparation of tetra-substituted pyrazoles **11**–**20**.

After the intermediates were prepared, ten tetra-substituted pyrazoles were synthesized, including two novel compounds, and four additional compounds were mentioned, but their physical properties were not specified. Specifically, the central framework of tetra-substituted pyrazoles is known, and other derivatives have been reported in the literature, such as those substituted with phenylamino in position 5 of the pyrazole ring [32]; however, these compounds are different from those obtained in this study. The synthesis involved nucleophilic addition reactions using hydrazine hydrate, methylhydrazine, phenylhydrazine, and thiosemicarbazide, which led to heterocyclization through microwave irradiation for 30 min (Scheme 6). The observed yields for the tetra-substituted pyrazoles (compounds **11**–**20**) ranged from 68.4% to 90.1%. In this study, the proper treatment of the metrics in the context of green chemistry, e.g., the E-factor and reaction time comparison, was not considered; however, we can highlight the use of microwave irradiation as an alternative energy source.

Infrared spectra revealed absorption bands common to the ten pyrazoles obtained. Thus, it was possible to observe a set of relatively thin bands whose intensities varied from medium to high, located between 730 and 600 cm^−1^, indicating the presence of the *S*-CH_3_ group. The remaining absorptions followed the expected pattern due to the nature of the structural groups.

In the ^1^H NMR spectra, singlets were observed in the range of 2.72 to 2.34 ppm, corresponding to the S-CH_3_ groups; 3.67 to 3.74 ppm assigned to O-CH_3_ of the ester group attached on C-4 of pyrazoles **11**, **12**, and **13**; and 3.98 ppm assigned to N-CH_3_ of pyrazole **17**. The chemical shifts of 6.27 and 6.41 ppm were attributed to NH_2_ attached to C-5 of pyrazoles **12** and **13**, respectively, while 9.14 and 9.76 ppm were attributed to NH_2_ on C-5 and thioamide attached to N-1, respectively, of pyrazole **11**. The chemical shifts of aromatic hydrogens were observed as multiplets in the range of 7.31 to 7.72 ppm of pyrazoles **14**–**17** and **20**.

The ^13^C NMR spectra showed chemical shifts assigned to carbon atoms of pyrazole rings of 70.9 to 93.8 ppm of C-4, 138.6 to 155.9 ppm of C-5, and 145.9 to 151.9 ppm of C-3. The chemical shifts assigned to the CH_3_-S groups ranged from 12.8 to 18.6 ppm. The other signals were attributed as expected.

### 2.2. Biological Predictions and In Vitro Assays

Before conducting biological tests, the molecular structures of pyrazole compounds were analyzed using the CLC-Pred [33] software to evaluate their cytotoxicity against non-tumor cells [34]. Compounds **11**, **12**, **13**, **17**, **19**, and **20** exhibited a low probability of being active (*Pa* values), ranging from 0.2070 in fibroblast cells to 0.3710 in umbilical vein endothelial cells, as shown in Table 1. This indicates that these compounds are unlikely to be active against non-tumor cells.

**Figure 1:**
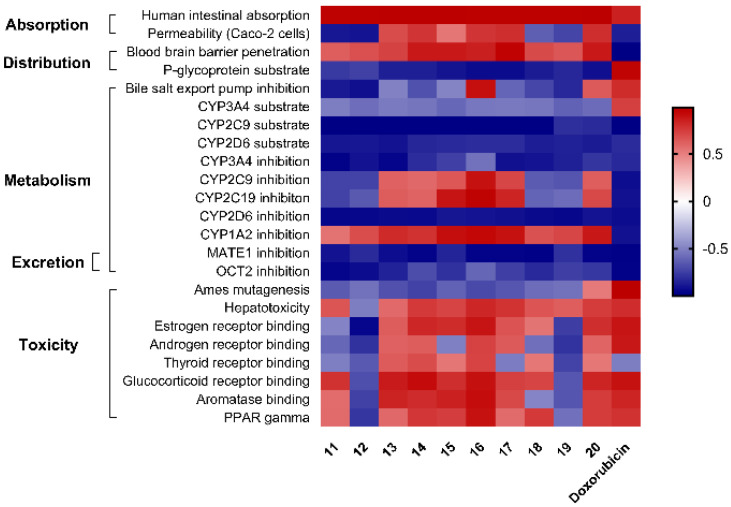
Predicted ADMET properties: AdmetSAR is a tool used to predict the absorption, distribution, metabolism, excretion, and toxicity properties of drug candidates. Doxorubicin values were used as a positive control.

The AdmetSAR software [35] was the second tool utilized in the in-silico analysis of pyrazoles **11**–**20**. This software can identify structural similarities between the studied molecules and others with known biological activity. Furthermore, it offers crucial predictions about the ADMET properties of these compounds (Figure 1).

The results from AdmetSAR indicated that all the pyrazoles synthesized in this study demonstrate good intestinal absorption and can effectively cross the blood–brain barrier, as evidenced by values exceeding 0.5 in both cases. None of the pyrazole compounds are expected to act as substrates for P-glycoprotein (ABCB1), indicating that tumor cells are unlikely to employ this resistance mechanism when these compounds are used as chemotherapeutic agents. Among the pyrazoles, only compounds **16** and **18** demonstrated potential for inhibiting the bile salt transporter, with the inhibitory value of pyrazole **16** being comparable to that of doxorubicin. Based on a brief analysis of the structure–activity relationship (SAR) using the free web server ADME [36], and considering the parameters of lipophilicity (logP), number of H-acceptors, number of H-donors, number of rotatable bonds, molar refractivity (RM), and topological polar surface area (TPSA) as summarized in Table S1 in the Supplementary Material, it can be seen that there is a better relationship between logP and TPSA for compounds **11** and **18**. The substituent groups that favored the best results were thiocarbonylamide at position 2 and NH_2_ at position 5 of the pyrazole ring.

**Figure 2:**
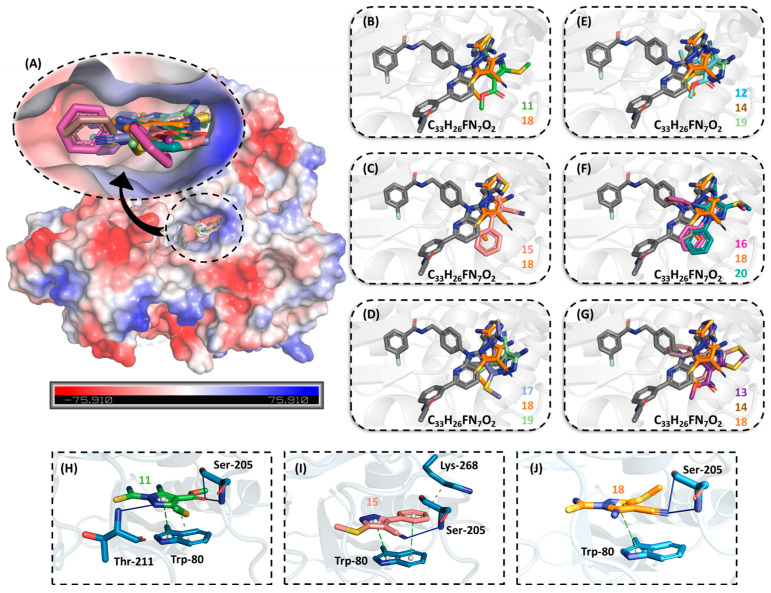
(**A**) The electrostatic potential map for AKT1 (PDB code: 4EJN) [36] with the superposition of the best docking pose to the pyrazole compounds **11**–**18**. Superposition between the crystallographic inhibitor C_33_H_26_FN_7_O_2_ (in black) with (**B**) **11**, **18**, (**C**) **15**, **18**, (**D**) **17**, **18**, **19**, (**E**) **12**, **14**, **19**, (**F**) **16**, **18**, **20**, and (**G**) **13**, **14**, **18** into the catalytic site of AKT1. The main amino acid residues with the corresponding interactive forces for (**H**) AKT1:**11**, (**I**) AKT1:**15**, and (**J**) AKT1:**18**. Blue lines and black and green dots mean hydrogen bond, hydrophobic, and π-stacking interactions.

The literature contains extensive reports about the main cancer-related targets for pyrazole derivatives [24,25,26,27], such as epidermal growth factor receptor (EGFR, kinase domain), vascular endothelial growth factor receptor 2 (VEGFR2, juxtamembrane and kinase domains), serine/threonine-protein kinase (isoform AKT1), and phosphoinositide 3-kinase (PI3K, p110a subunit). To provide a molecular-level suggestion of feasible targets for the tetra-substituted pyrazole derivatives **11**–**20** as potential anti-tumor agents, molecular docking calculations were conducted on EGFR, VEGFR2, AKT1, and PI3K, with the docking scores summarized in Table 2. In the GOLD 2024.2 software, the docking score value (dimensionless) for each pose accounts for intramolecular tensions within the ligand and intermolecular interactions and is considered as the negative value of the sum of the energy terms involved in the macromolecule–ligand association; thus, the more positive the score, the better the interactive profile [37]. In this case, the positive docking score values obtained for all evaluated targets related to **11**–**20** suggest that the pyrazole derivatives may interact with different cancer-related proteins; however, AKT1 can be considered the main target due to its highest docking score value, e.g., 40.2, 36.9, 65.1, and 43.0 dimensionless for EGFR, VEGFR2, AKT1, and PI3K, respectively, for the pyrazole derivative **11** (Table 2). Furthermore, the highest docking score values were recorded for the pyrazole derivatives **11**, **13**, **14**, **15**, **17**, **18**, and **20**, indicating that these compounds are potential anti-tumor agents, fitting well into a positive electrostatic potential pocket (Figure 2A). The heterocyclic core of the crystallographic AKT1 inhibitor *N*-(4-(5-(3-acetamidophenyl)-2-(2-aminopyridin-3-yl)-3*H*-imidazo [4,5-*b*]pyridin-3-yl)benzyl)-3-fluorobenzamide (C_33_H_26_FN_7_O_2_) [38] mainly superposes with the pyrazole core of **11**, **15**, and **18** (Figure 2B–G), stabilized by hydrogen bonds, hydrophobic interactions, and π-stacking forces (Figure 2H–J). It is important to highlight that AKT1 should be considered as a target for pyrazole derivatives, mainly due to the vast biological recognition of the pyrazole core [12,39]. Thus, future experimental enzymatic assays are required to achieve a better understanding of the correlation between the chemical structure of **11**–**20** and their anticancer profile.

To validate the in-silico predictions, pyrazoles **11** and **18** were selected for experimental biological assays. These compounds were chosen due to their favorable ADME properties, exhibiting low toxicity or toxicity comparable to doxorubicin, and demonstrating good docking scores to AKT1, with superposition of the heterocyclic core of the crystallographic inhibitor C_33_H_26_FN_7_O_2_.

The assays to evaluate the general toxicity of pyrazoles **11** and **18** were based on their toxic capacity on the species of *Artemia salina*, a microcrustacean, through a quick and low-cost method [35]. The observed survival percentages were 99% and 100%, respectively, at 200 μM, indicating that these compounds did not affect the viability of brine shrimp and that they were not toxic to this species, in line with the toxicity predictions.

**Figure 3:**
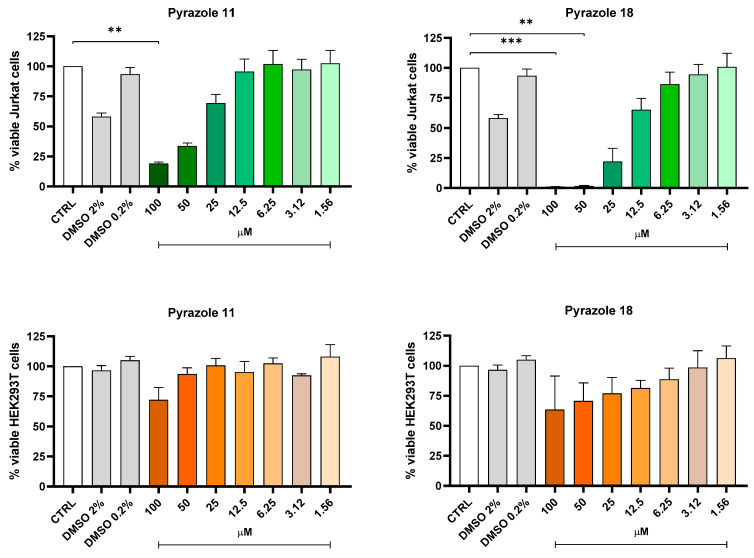
The effects of pyrazole on the cellular viability of Jurkat (green graphics) and HEK293 (orange graphics). The 2 × 10^5^ cells/mL were incubated with or without DMSO (0.2%) and pyrazole **11** or **18** at concentrations in the range of 1.56–100 µM. After 72 h at 37 °C in a humidified atmosphere with 5% CO_2_, cell viability was assessed using the MTT assay. Cell viability percentage was calculated considering the cells cultured with medium (CTRL) as 100% viable cells. The data are presented as mean values ± standard error of the mean (SEM) from triplicate independent experiments (n = 2 or 3). ** *p* = 0.008 *** *p* = 0.0001 were considered statistically significant.

Thus, the treatment of leukemic cells of the Jurkat lineage was carried out with the selected compounds at concentrations in the range of 1.56–100 µM. Figure 3 depicts the cell viability results for the evaluated compounds.

Considering the cytotoxicity on Jurkat cells, as summarized in Figure 3, pyrazole **11** had a half-maximal inhibitory concentration (IC_50_) value of 45.05 µM, while pyrazole **18** exhibited an IC_50_ value of 14.85 µM. These values indicate that the effects on Jurkat cell viability are more promising for pyrazole **18** than for pyrazole **11**, agreeing with the obtained docking score values. Analyzing their respective molecular structures, there is a change only in the substituent linked to the pyrazole ring at position 4, namely, a methyl ester group at **11** and a nitrile at **18**. Thus, it is possible to suggest that the nitrile group at position 4 of the pyrazole ring exhibits significant potential for cytotoxic effects against Jurkat cells when compared to an ester group in the same position. Furthermore, assays were performed with the HEK 293 cell line (non-carcinogenic cells, Figure 3), and cell viability results indicated that the IC_50_ values for **11** and **18** were above 100 µM, clearly showing selectivity of the pyrazole derivatives under study to cancer cell lines, which corroborates with the cytotoxicity prediction using CLC-Pred software [33] (Table 1). It is important to highlight that although the reported doxorubicin exhibits better potency than the evaluated pyrazoles (around 40 nM) [40], its cytotoxic activity is accompanied by nonspecific effects, including gastrointestinal and urogenital concerns, cardiotoxicity, and neurotoxicity [41]. In this sense, the obtained pyrazole derivatives might be considered pharmacologically relevant. In future assays, other carcinogenic cell lines will be evaluated to complement the preliminary results obtained in this study.

## 3. Materials and Methods

### 3.1. Chemicals and Instruments

Solvents and reagents were purchased from Merck KGaA company (Darmstadt, Germany) and used without purification. The nitrile intermediates **4**–**6** were synthesized according to the published procedures [28,29,30,31].

The NMR spectra were acquired on a spectrometer on a Bruker NMR Ultrashield 500 MHz (BrukerBioSpin GmbH, Rheinstetten, Germany), and tetramethylsilane and DMSO-d_6_ were used as internal reference and solvent, respectively. Chemical shifts (δ) are mentioned as ppm. The Fourier transform infrared (FT-IR) spectra were obtained using a Bruker Vertex 70 (BrukerBioSpin GmbH, Rheinstetten, Germany), and the melting points were measured using Gehaka PF1500 (São Paulo, Brazil). High-resolution mass spectra of new compounds were recorded on a Bruker Compass Data Analysis 4.2 by electrospray ionization (BrukerBioSpin GmbH, Rheinstetten, Germany). Thin-layer chromatography (TLC, a fine-sheet chromatography of silica gel 60) was used to monitor the reactions, and the spots were revealed by exposure to UV light (254 and 365 nm). The scientific microwave oven used in the pyrazole synthesis was Anton Paar Monowave 300 (Los Angeles, CA, USA).

### 3.2. General Procedure for the Synthesis of Tetra-Substituted Pyrazoles *(**11**–**20**)*

In a scientific microwave tube containing 3 mL of ethanol, an equimolar solution of each nitrile precursor (**4**–**6**) was prepared alongside one of four nucleophiles: thiosemicarbazide (**7**), hydrazine hydrate (**8**), phenylhydrazine (**9**), and methylhydrazine (**10**). Following the homogenization of the solution, the tube was sealed and placed into a scientific microwave oven, which was set to a temperature of 80 °C for thirty minutes. The reaction’s progress was monitored using TLC with a developing solvent mixture of dichloromethane and ethyl acetate (2:1, *v*/*v*). The resulting products were collected in solid form through vacuum filtration, washed with ice-cold ethanol, and then purified via recrystallization from ethanol. The spectra used to characterize the organic compounds are provided as Figures S1–S33 in the Supplementary Material.

*Methyl 5-amino-1-carbamothioyl-3-methylsulfanyl-1H-pyrazol-4-carboxylate* (**11**): Yield 83%; light yellow solid; mp 189–191 °C; FTIR (ATR) ν/cm^−1^ 3450, 3410, 3315, 3270, 1860, 1300, 700 cm^−1^; ^1^H NMR (500 MHz, DMSO-d_6_) δ 9.76 (s, 2H), 9.14 (s, 2H), 3.73 (s, 3H), 2.46 (s, 3H); ^13^C NMR (125 MHz, DMSO-d_6_) δ 176.6, 163.4, 155.0, 151.9, 128.0, 92.5, 51.3; HRMS (EIS) *m*/*z* [M + H]^+^ calcd. for C_7_H_12_N_4_O_2_S_2_^+^: 247.0318; found: 247.0342.

*Methyl 5-amino-3-methylsulfanyl-1H-pyrazol-4-carboxylate* (**12**): Yield 86%; brown solid; mp 171–174 °C; FTIR (ATR) ν/cm^−1^ 3450, 3300, 1750, 1350, 700 cm^−1^; ^1^H NMR (500 MHz, DMSO-d_6_) δ 6.27 (s, 1H), 3.67 (s, 2H), 2.34 (s, 3H); ^13^C NMR (125 MHz, DMSO-d_6_) δ 164.1, 153.4, 147.6, 12.9, 91.9, 50.7; HRMS (EIS) *m*/*z* [M + H]^+^ calcd. for C_6_H_10_N_3_O_2_S^+^: 188.0488; found: 188.0504.

*Methyl 5-amino-1-phenyl-3-methylsulfanyl-1H-pyrazol-4-carboxylate* (**13**): Yield 75%; pearly white solid; mp 118–120 °C (close to the reported ethyl ester, 95–97 °C) [42]; FTIR (ATR) ν/cm^−1^ 3340, 3320, 3100, 1680, 1600, 1460, 700 cm^−1^; ^1^H NMR (500 MHz, DMSO-d_6_) δ 7.55 (d, 4H, *J* 3.9 Hz), 7.54 (m, 1H), 6.41 (s, 2H), 3.74 (s, 3H), 2.40 (s, 3H); ^13^C NMR (125 MHz, DMSO-d_6_) δ 164.0, 151.4, 149.3, 138.2, 129.9, 128.8, 128.0, 124.2, 92.9, 51.1.

*4-Cyano-3-methylsulfanyl-5-phenyl-1H-pyrazol-1-carbothioamide* (**14**): Yield 72%; yellow solid; mp 123–127 °C; FTIR (ATR) ν/cm^−1^ 34600, 3280, 2300, 1600, 1500, 700 cm^−1^; ^1^H NMR (500 MHz, DMSO-d_6_) δ 7.72–7,45 (m, 5H), 2.36 (s, 3H); ^13^C NMR (125 MHz, DMSO-d_6_) δ 164.0, 151.4, 149.3, 138.2, 129.9, 128.0, 124.2, 92.9, 51.1, 12.8; HRMS (EIS): *m*/*z* [M + H - HNCS]^+^ calcd. for [C_12_H_11_N_4_S_2_–HNCS]^+^: 214.0445; found: 214.0406.

*3-Methylsulfanyl-5-phenyl-1H-pyrazol-4-carbononitrile* (**15**): Yield 81%; beige solid; mp 152–153 °C (147–149 °C) [43]; FTIR (ATR) ν/cm^−1^ 2300, 1600, 1550, 700 cm^−1^; ^1^H NMR (500 MHz, DMSO-d_6_) δ 11.63 (s, 1H), 7.62 (m, 5H), 2.72 (s, 3H); ^13^C NMR (125 MHz, DMSO-d_6_) δ 151.4, 148.3, 139.3, 129.0, 128.1, 127.8, 127.5, 127.3, 117.0, 88.9, 12.8.

*5-Methylsulfanyl-1,3-diphenyl-4-carbonitrile* (**16**): Yield 69%; pinkish white solid; mp 115–117 °C (119–120 °C) [44]; FTIR (ATR) ν/cm^−1^ 3100, 2225, 1600, 1450, 655 cm^−1^; ^1^H NMR (500 MHz, DMSO-d_6_) δ 7.47–7.34 (m, 10H), 2.65 (s, 3H); ^13^C NMR (125 MHz, DMSO-d_6_) δ 151.3, 149.7, 138.6, 130.9, 129.7, 129.6, 129.5, 126.7, 126.1, 113.7, 92.8, 14.5.

*5-Methylsulfanyl-1-methyl-3-phenyl-1H-pyrazol-4-carbonitrile* (**17**): Yield: 91%; ocher-yellow solid; mp 85–89 °C; FTIR (ATR) ν/cm^−1^ 3100, 2225, 1650, 1500, 655 cm^−1^; ^1^H NMR (500 MHz, DMSO-d_6_) δ 7.86–7.48 (m, 5H), 3.98 (s, 3H), 2.60 (s, 3H); ^13^C NMR (125 MHz, DMSO-d_6_) δ 151.8, 144.1, 130.7, 130.0, 126.4, 114.9, 93.8, 38.1, 18.4, 12.6; HRMS (EIS) *m*/*z* [M + H]^+^ calcd. for C_12_H_12_N_3_S^+^: 230.0746; found: 230.0756.

*5-Amino-4-cyano-3-methylsulfanyl-1H-pyrazol-1-carbothioamide* (**18**): Yield 74%; yellow solid; mp 147–151 °C; FTIR (ATR) ν/cm^−1^ 3340, 3250, 3200, 3175, 2205, 730 cm^−1^; ^1^H NMR (500 MHz, DMSO-d_6_) δ 8.52 (s, 2H), 6.95 (s, 2H), 2.54 (s, 3H); ^13^C NMR (125 MHz, DMSO-d_6_) δ 181.2, 150.0, 145.9, 113.4, 76.1, 18.6, 50.7; HRMS (EIS) *m*/*z* [M + H]^+^ calcd. for C_6_H_8_N_5_S_2_^+^: 214.0227; found: 214.0256.

*5-Amino-3-methylsulfanyl-1H-pyrazol-4-carbonitrile* (**19**): Yield 68%; yellow solid; mp 149–150 °C (152 °C) [45] FTIR (ATR) ν/cm^−1^ 3295, 3127, 2290, 700 cm^−1^; ^1^H NMR (500 MHz, DMSO-d_6_) δ 12.00 (s, 1H), 6.41 (s, 2H), 2.43 (s, 3H); ^13^C NMR (125 MHz, DMSO-d_6_) δ 155.1, 149.2, 115.3, 76.7, 14.3.

*5-Amino-3-methylsulfanyl-1-phenyl-1H-pyrazol-4-carbonitrile* (**20**): Yield: 77%; translucid solid; mp 129–134 °C (136 °C) [45,46]; FTIR (ATR) ν/cm^−1^ 3450, 3330, 2305, 1600, 1550, 700 cm^−1^; ^1^H NMR (500 MHz, DMSO-d_6_) δ 7.47 (m, 5H), 6.70 (s, 2H), 2.46 (s, 3H); ^13^C NMR (125 MHz, DMSO-d_6_) δ 152.7, 149.5, 137.4, 130.6, 128.6, 124.6, 114.5, 73.9, 13.8.

### 3.3. In-Silico Prediction of Biological Activity

CLC-Pred and AdmetSAR software (version 2.0) [33,35] was utilized to evaluate the toxicity potential and pharmacological parameters in silico, using the method of representing chemical structures called SMILES. Additionally, the physicochemical properties and lipophilicity were obtained from the web server SwissADME [36].

### 3.4. Molecular Docking Calculations

The three-dimensional structures of the cancer-related targets were obtained in the Protein Data Bank (PDB), with access codes 2ITY [47], 4ASD [48], 4EJN [38], and 4TV3 [49]. The chemical structure of the tetra-substituted pyrazoles **11**–**20** was built and energy-minimized with Spartan software version 18 (Wavefunction, Inc., Irvine, CA, USA) [50]. On the other hand, molecular docking calculations were performed with GOLD 2024.3 software (Cambridge Crystallographic Data Centre, Cambridge, CB2 1EZ, UK) [37], considering an 8 Å radius around the active site and using ChemPLP as the scoring function due to the obtained lowest root mean square deviation (RMSD) values to the heterocyclic crystallographic AKT1 inhibitor *N*-(4-(5-(3-acetamidophenyl)-2-(2-aminopyridin-3-yl)-3*H*-imidazo [4,5-*b*]pyridin-3-yl)benzyl)-3-fluorobenzamide (C_33_H_26_FN_7_O_2_) (Figure S34 in the Supplementary Material), agreeing with our previous studies [51,52]. The web server Protein-Ligand Interaction Profiler (PLIP) [53] was used to identify the main amino acid residues that interact with tetra-substituted pyrazoles **11**–**20**, while PyMOL Molecular Graphics System 1.0 level software (Delano Scientific LLC software, Schrodinger, New York, NY, USA) [54] was used to generate docking representations.

### 3.5. Artemia Salina Assays

The toxicity assays with *Artemia salina* were carried out according to the methodology described by Meyer and collaborators [55]. An artificial saline solution was prepared at a concentration of 30 g·L^−1^, and the pH was adjusted between 8 and 9 [56]. The cysts (3 g) were placed in six-well plates containing 5 mL of the solution per well, keeping them at room temperature with partial lighting and aeration for a period of 24h. After this time, the nauplii were transferred to 24-well plates in the presence or absence of the compounds. The tests with pyrazoles were carried out in duplicate at four different concentrations: 25, 50, 100, and 200 µM. As controls, nauplii were kept only in saline solutions, and nauplii were treated with a mixture of saline and DMSO (0.4 and 4%, respectively). After this, the survival of the nauplii was assessed, with observations carried out using a stereoscopic microscope. Those without internal or external movements for 10 s were considered dead nauplii [57]. Using Equation (1), based on the relationship between the number of dead nauplii observed after the incubation period and the total number of nauplii placed in the well, it was possible to calculate the amount of a chemical that is lethal to one-half (lethal dose 50%, LD_50_). Values below 25 µM are considered to indicate low toxicity, while values above 100 µM are considered very toxic [58].

Mortality rate = (number of dead nauplii)/(total number) × 100 (1)

### 3.6. Cell Viability Assays

Jurkat cells, a lineage of leukemic T cells, were cultivated at a concentration of 2 × 10^5^ cells/mL, and Human Embryonic Kidney (HEK) 293 at a 4 × 10^5^ cells/mL. The cells were maintained in plastic bottles suitable for cell culture using Roswell Park Memorial Institute medium (RPMI for Jurkat) or Dulbecco’s Modified Eagle Medium (DMEM for HEK293), both supplemented at 10% of the volume with inactivated fetal bovine serum and 100 U/mL of penicillin-streptomycin, which was used to cultivate the cells. Jurkat and HEK293 cells were incubated in triplicate at 37 °C in a humidified atmosphere with 5% CO_2_ in the presence or absence of pyrazoles (1.56 to 100 µM). Controls were prepared from cells treated with DMSO (0.2%) and cells treated with medium alone. Cell viability was analyzed using the 3,4,5-dimethylthiazol-2-yl-2,5-diphenyltetrazolium bromide (MTT) assay after a period of 72h of treatment with pyrazoles. In this case, 20 µL of MTT at a concentration of 5 mg/mL was added to each well and incubated for 3 h. At the end of this time, the cells were centrifuged at 250× *g*, the supernatant discarded, and the formazan crystals dissolved in 200 µL of DMSO. Finally, using a spectrophotometer for microplates (SpectraMax i3, Molecular Devices, Wals, Austria), a reading was taken at a wavelength of 490 nm.

### 3.7. Statistical Analysis

Statistical analysis was performed by one-way analysis of variance (ANOVA), and the means were compared using a Tukey post-test. The IC_50_ values were obtained using nonlinear regression. Experimental data were analyzed in graphs using GraphPad Prism 8.0 software. *p* < 0.05 values were considered significant.

## 4. Conclusions

The three nitrile precursors proved to be important units for the construction of the pyrazole ring, and together with the nucleophiles used (hydrazines and thiosemicarbazide), these building blocks resulted in a series of molecules with a diverse group of substituents attached to the heterocyclic ring. The methodology employed for the synthesis of the tetra-substituted pyrazole nucleus, using the microwave oven, proved to be an approach that meets some of the principles of green chemistry, such as the use of alternative energy and the absence of toxic solvents. The spectroscopic methods used to characterize the compounds confirmed the proposed structures. The ADMET prediction, combined with molecular docking calculations for four main cancer-related targets to pyrazoles, proved to be a crucial tool for saving time in vitro assays. These tools allowed for the screening of pyrazoles, increasing the likelihood of identifying compounds that exhibit biological activity while minimizing toxicity. Cell viability assays demonstrated that both tested pyrazoles significantly affected the Jurkat cell line (IC_50_ values of 45.05 µM and 14.85 µM to **11** and **18**, respectively), corroborating the findings from the in-silico predictions. Finally, the assayed compounds showed selectivity against cancer cell lines due to potency above 100 µM against the non-carcinogenic cells HEK 293.

## Figures and Tables

**Table 1:** Prediction of low cytotoxicity against non-tumor cells.

Compounds	*Pa* ^1^	Non-Tumor Cell Lineage Prediction Results
**11**	0.2110	BJ (foreskin fibroblast)
**12**	0.2070	HEL299 (fibroblast)
**13**	0.2400	BJ (foreskin fibroblast)
**17**	0.2120	HEL299 (fibroblast)
**19**	0.2190	BJ (foreskin fibroblast)
**20**	0.2070	HEL299 (fibroblast)

^1^ Probability of being active.

**Table 2:** Docking score values (dimensionless) for the interaction between tetra-substituted pyrazoles **11**–**20** and selected cancer-related targets.

Compounds	2ITY	4ASD	4EJN	4TV3
**11**	40.2	36.9	65.1	43.0
**12**	32.9	32.2	43.3	40.4
**13**	42.5	60.3	67.3	58.0
**14**	41.9	48.5	63.5	53.8
**15**	38.8	52.6	67.2	52.1
**16**	51.5	52.0	46.1	62.9
**17**	43.8	50.6	64.4	50.8
**18**	32.5	39.8	68.4	40.7
**19**	26.7	30.2	39.6	36.9
**20**	43.1	52.7	63.7	58.1

## Data Availability

All the raw data obtained in this study will be available upon request.

## Supplementary Materials

The following supporting information can be downloaded at: https://www.mdpi.com/article/10.3390/molecules30132880/s1, Figures S1–S3. FT-IR spectra of nitrile intermediates **4**–**6**. Figures S4–S13. FT-IR spectra of tetra-substituted pyrazoles **11**–**20**. Figures S14–S23. ^1^H NMR spectra in DMSO-d_6_ of tetra-substituted pyrazoles **11**–**20**. Figures S24–S33. ^13^C NMR spectra in DMSO-d_6_ of tetra-substituted pyrazoles **11**–**20**. Figure S34. Superposition of the heterocyclic crystallographic AKT1 inhibitor *N*-(4-(5-(3-acetamidophenyl)-2-(2-aminopyridin-3-yl)-3*H*-imidazo [4,5-b]pyridin-3-yl)benzyl)-3-fluorobenzamide (C_33_H_26_FN_7_O_2_, PDB code 4EJN) and its corresponding best redocking pose using the molecular docking functions ChemPLP, ChemScore, GoldScore, and ASP. The root mean square deviation (RMSD) values for each docking function were highlighted with the corresponding color used in the stick representation. The crystallographically reported structure of C_33_H_26_FN_7_O_2_ is shown in black stick representation. To facilitate interpretation, hydrogen atoms were omitted. Table S1. Physicochemical properties and lipophilicity obtained from the web server SwissADME.
