# Supplementary material for: Synthesis of Novel Tetra-Substituted Pyrazole Derivatives Using Microwave Irradiation and Their Anti-Leukemic Activity Against Jurkat Cells
Source: Molecules. 2025 Jul 7;30(13):2880. doi: 10.3390/molecules30132880 (PMC12251036; doi:10.3390/molecules30132880)
Supplement: Supplementary file 1 [file molecules-30-02880-s001.zip › molecules-3701036-supplementary.pdf]

Supplementary Material

Synthesis of Novel Tetra-Substituted Pyrazole Derivatives Using Microwave Irradiation and Their Anti-Leukemic Activity Against Jurkat Cells

Felipe P. Machado [1], Maria Clara Campos [2], Juliana Echevarria-Lima [2], Diego P. Sangi [3], Carlos Serpa [4], Otávio Augusto Chaves [4,5,*] and Aurea Echevarria [1,*]

[1] Department of Organic Chemistry, Institute of Chemistry, Federal Rural University of Rio de Janeiro, Seropédica 23897-000, RJ, Brazil; felipepires@ufrrj.br
[2] Paulo de Góes Institute of Microbiology, Federal University of Rio de Janeiro, Rio de Janeiro 21941-902, RJ, Brazil; mariaclara.scampos@gmail.com (M.C.C.); juechevarria@micro.ufrj.br (J.E.-L.)
[3] Institute of Exact Sciences, Fluminense Federal University, Volta Redonda 27213-145, RJ, Brazil; dpsangi@id.uff.br
[4] Department of Chemistry, Coimbra Chemistry Centre—Institute of Molecular Science, University of Coimbra, Rua Larga, 3004-535 Coimbra, Portugal; serpasoa@uc.pt
[5] Laboratory of Immunopharmacology, Centro de Pesquisa, Inovação e Vigilância em COVID-19 e Emergências Sanitárias, Oswaldo Cruz Institute, Oswaldo Cruz Foundation, Rio de Janeiro 21040-361, RJ, Brazil
* Correspondence: otavioaugustochaves@gmail.com (O.A.C.); echevarr@ufrrj.br (A.E.)

Index

Figures S1-S3. FT-IR spectra of nitrile intermediates **4-6.**

Figures S4-S13. FT-IR spectra of tetra-substituted pyrazoles **11-20.**

Figures S14-S23. ^{1}H NMR spectra in DMSO-d_6 of tetra-substituted pyrazoles **11-20.**

Figures S24-S33. ^{13}C NMR spectra in DMSO-d_6 of tetra-substituted pyrazoles **11-20.**

Figure S34. Superposition of the heterocyclic crystallographic AKT1 inhibitor *N*-(4-(5-(3-acetamidophenyl)-2-(2-aminopyridin-3-yl)-3*H*-imidazo[4,5-b]pyridin-3-yl)benzyl)-3-fluorobenzamide ($C_{33}H_{26}FN_7O_2$, PDB code 4EJN) and its corresponding best redocking pose using the molecular docking functions ChemPLP, ChemScore, GoldScore, and ASP. The root mean square deviation (RMSD) values for each docking function were highlighted with the corresponding color used in the stick representation. The crystallographically reported structure of $C_{33}H_{26}FN_7O_2$ is in stick representation in black. For better interpretation, hydrogen atoms were omitted.

Table S1. Physicochemical properties and lipophilicity obtained from the web server SwissADME.

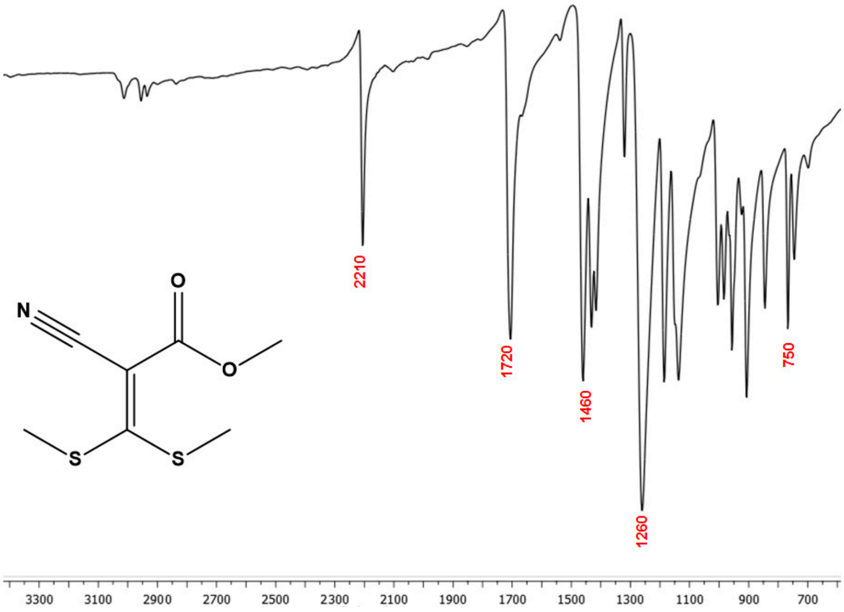

FT-IR spectra of compound **4**

Figure S1. FTIR (ATR) spectrum of intermediate **4.**

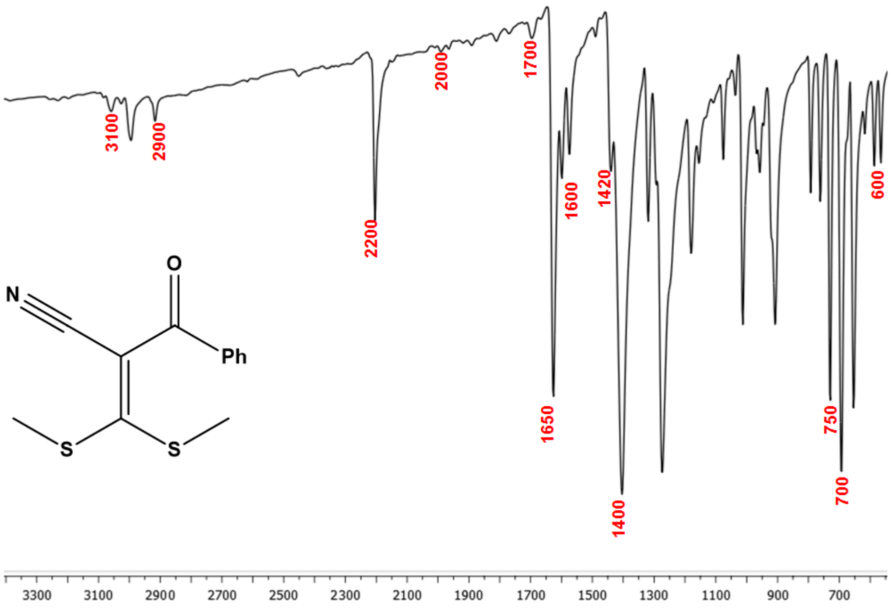

FT-IR spectra of compound **5**

Figure S2. FTIR (ATR) spectrum of intermediate **5.**

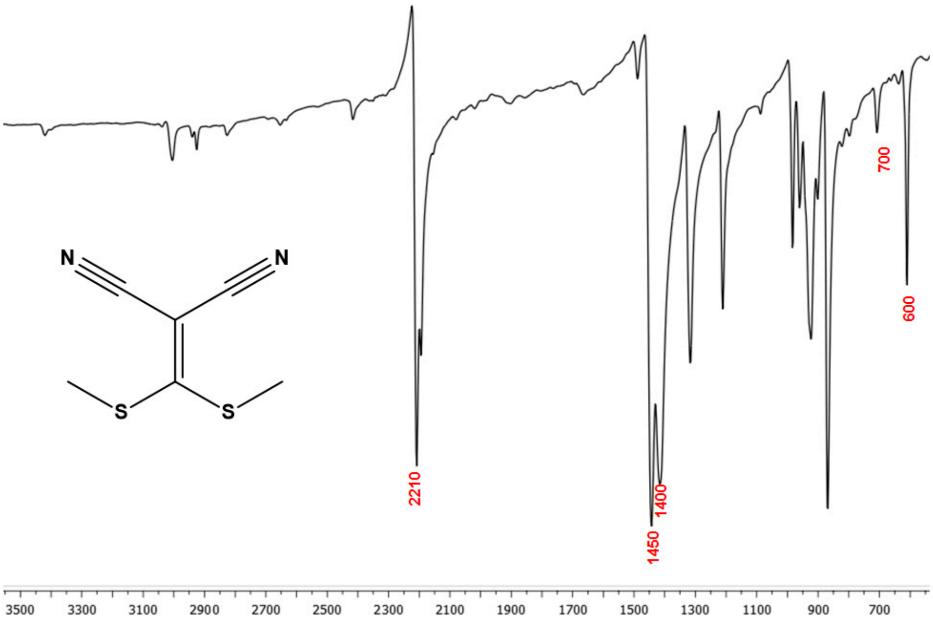

Figure S3. FTIR (ATR) spectrum of intermediate **6.**

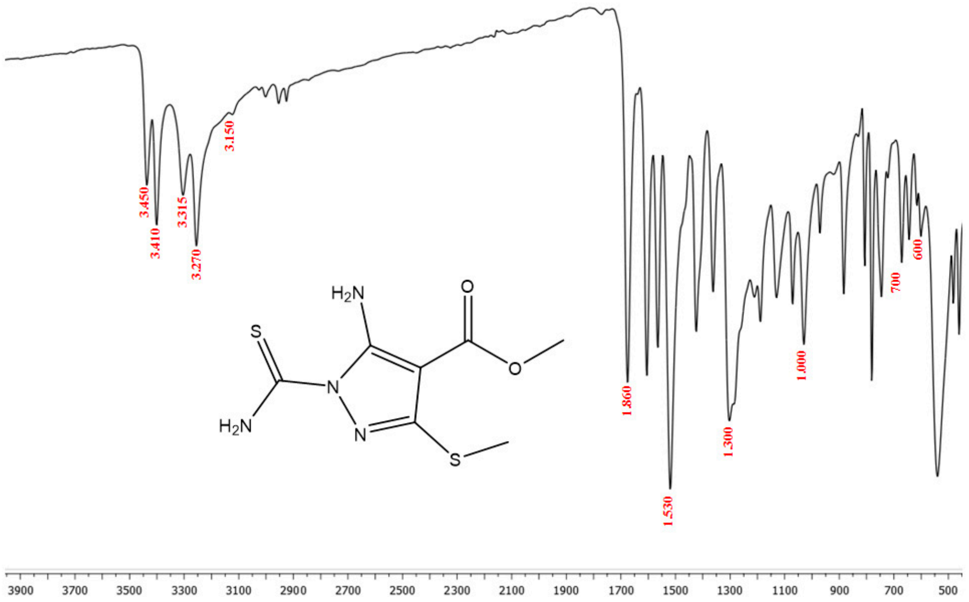

Figure S4. FTIR (ATR) spectrum of compound **11.**

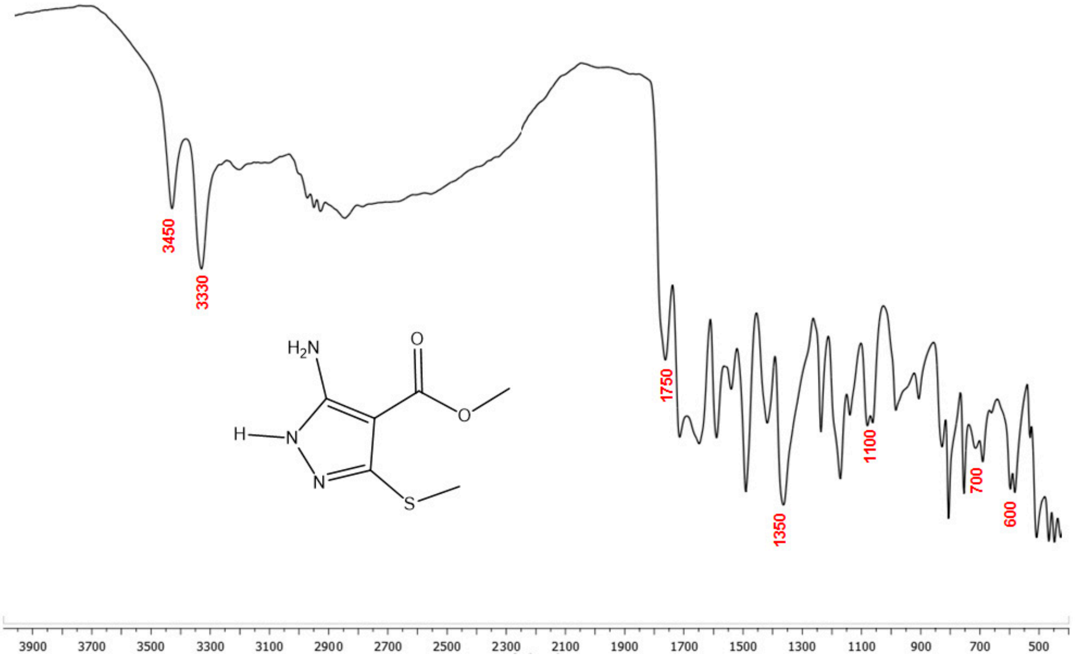

Figure S5. FTIR (ATR) spectrum of compound **12.**

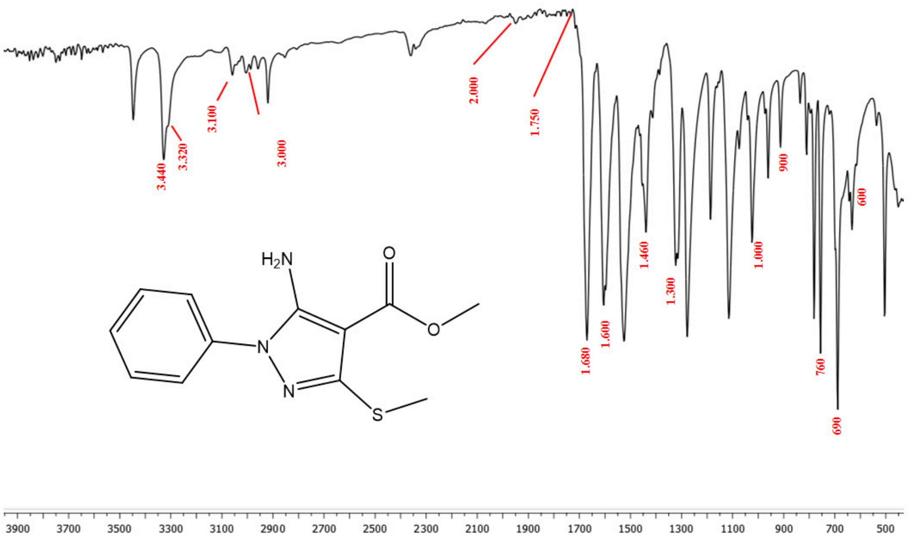

Figure S6. FTIR (ATR) spectrum of compound **13.**

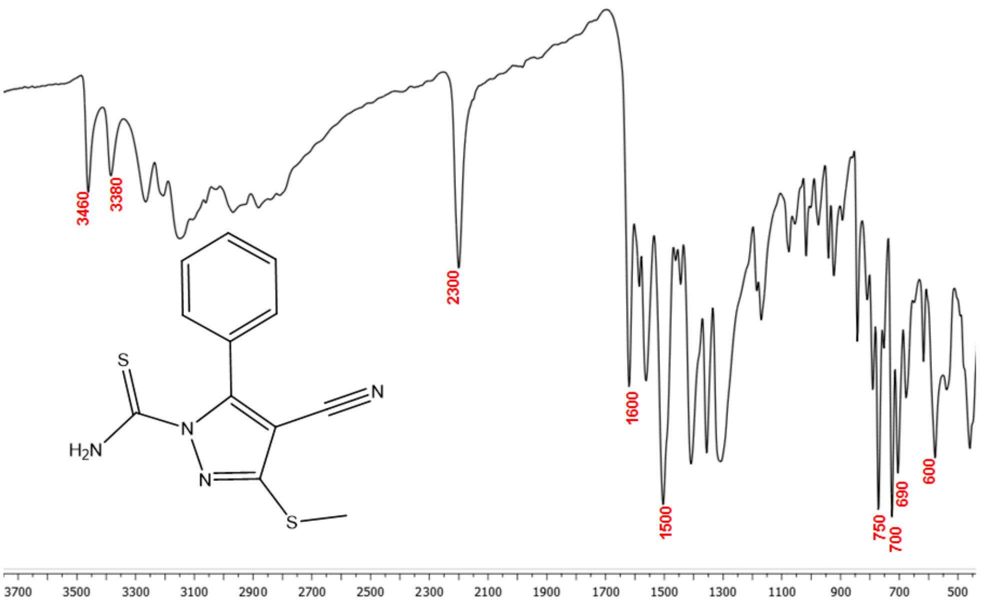

Figure S7. FTIR (ATR) spectrum of compound **14.**

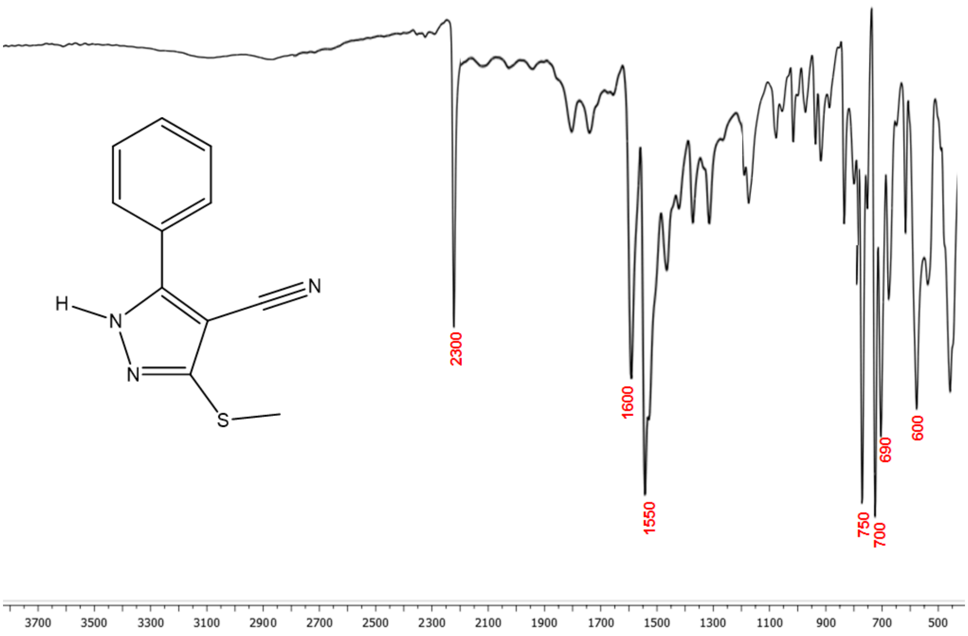

Figure S8. FTIR (ATR) spectrum of compound **15.**

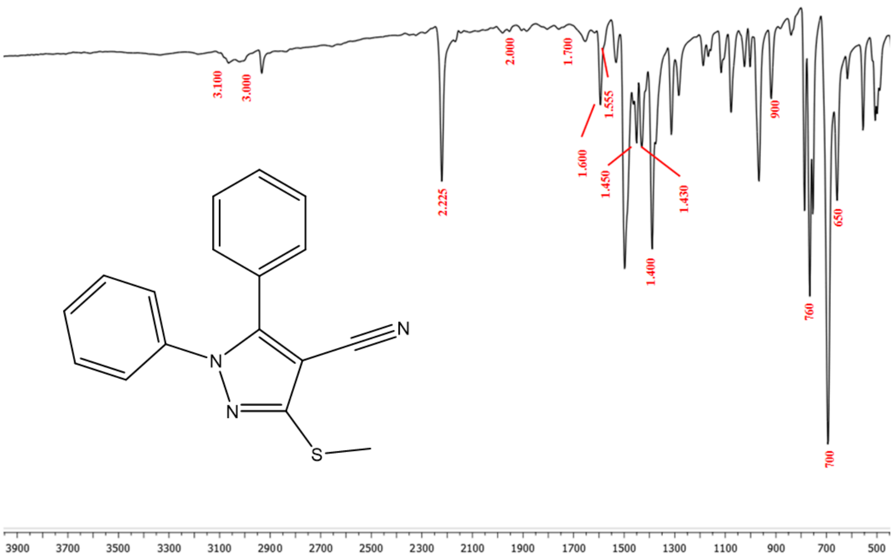

Figure S9. FTIR (ATR) spectrum of compound **16.**

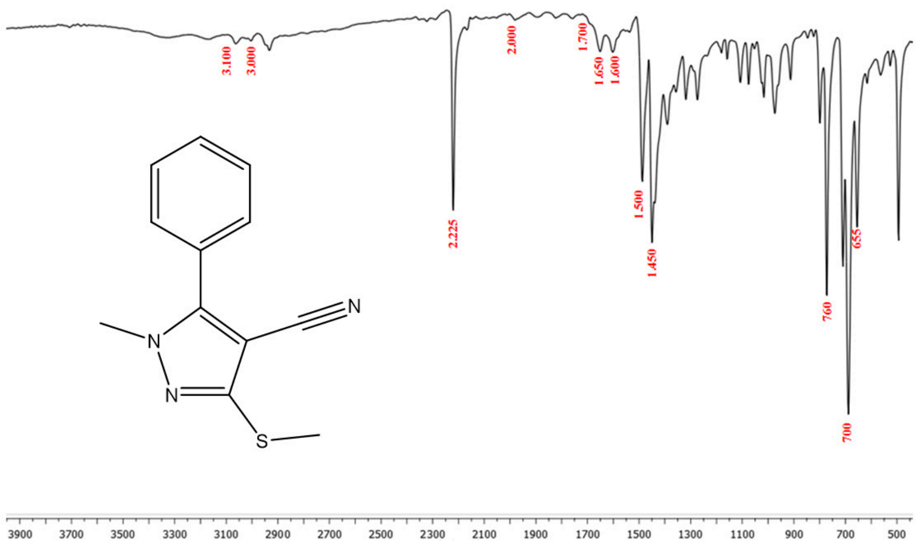

Figure S10. FTIR (ATR) spectrum of compound **17.**

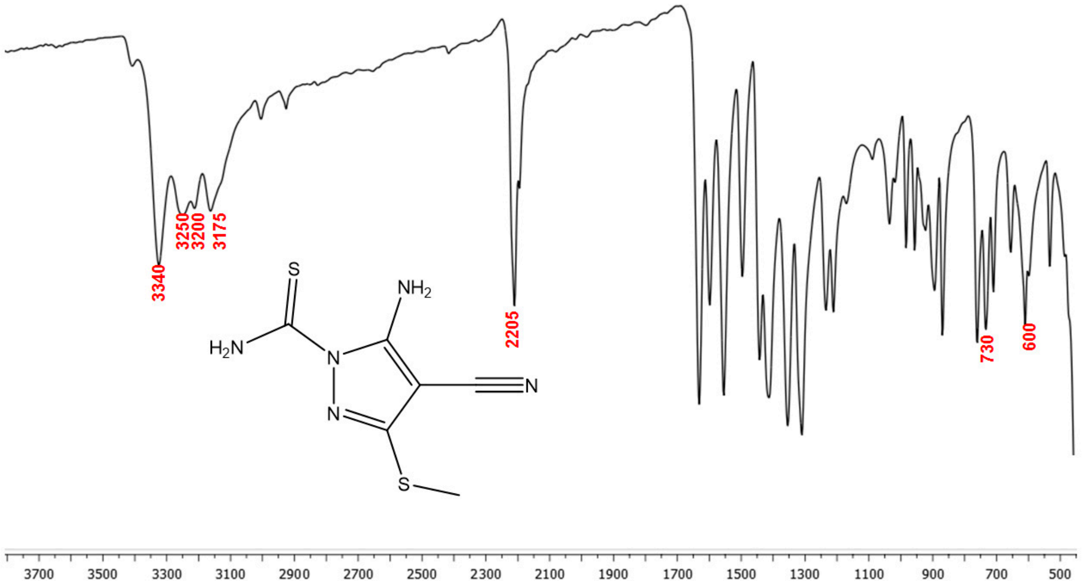

Figure S11. FTIR (ATR) spectrum of compound **18.**

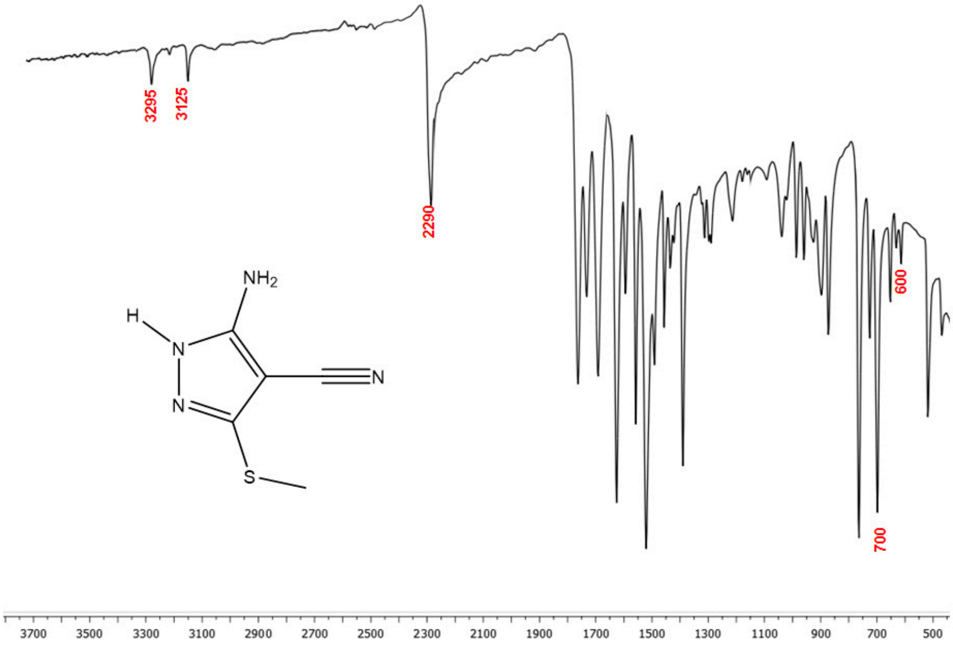

Figure S12. FTIR (ATR) spectrum of compound **19.**

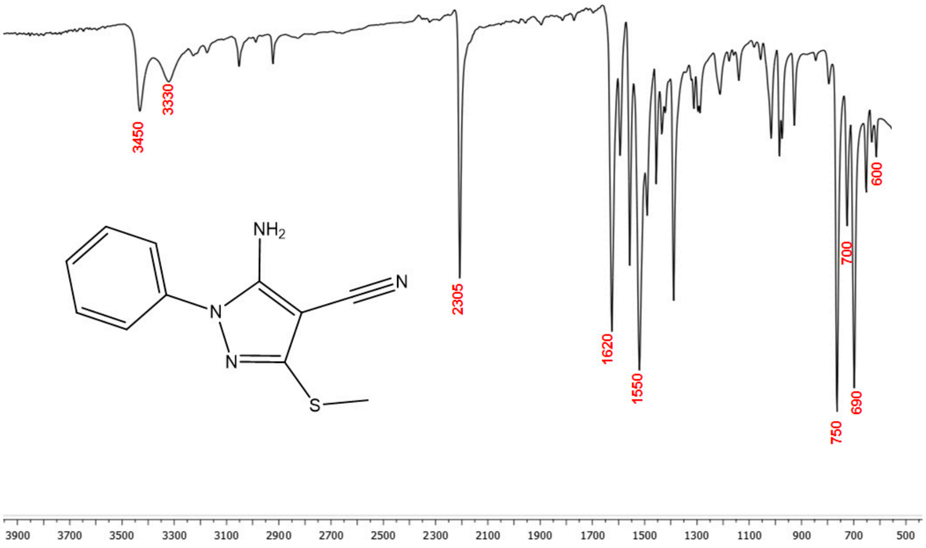

Figure S13. FTIR (ATR) spectrum of compound **20.**

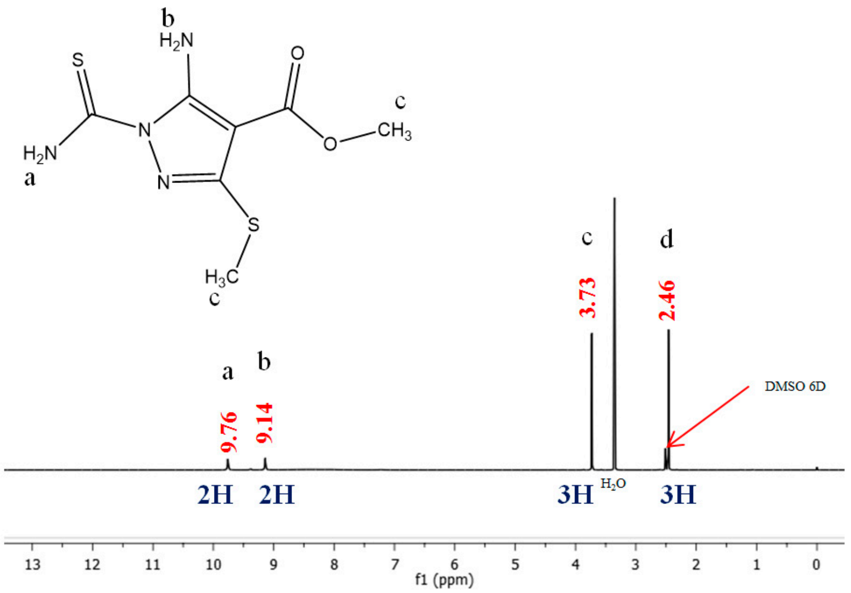

Figure S14. ^{1}H NMR spectrum (500 MHz, DMSO-d_6) of compound **11**

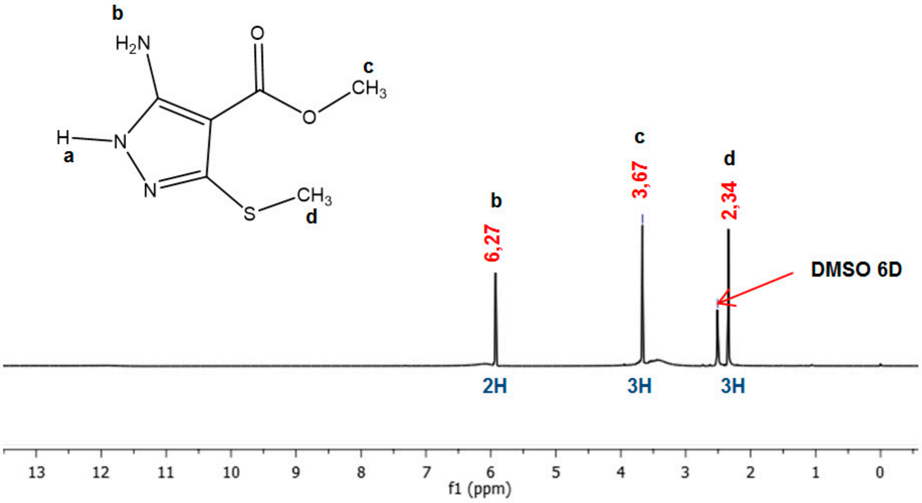

Figure S15. ^{1}H NMR spectrum (500 MHz, DMSO-d_6) of compound **12.**

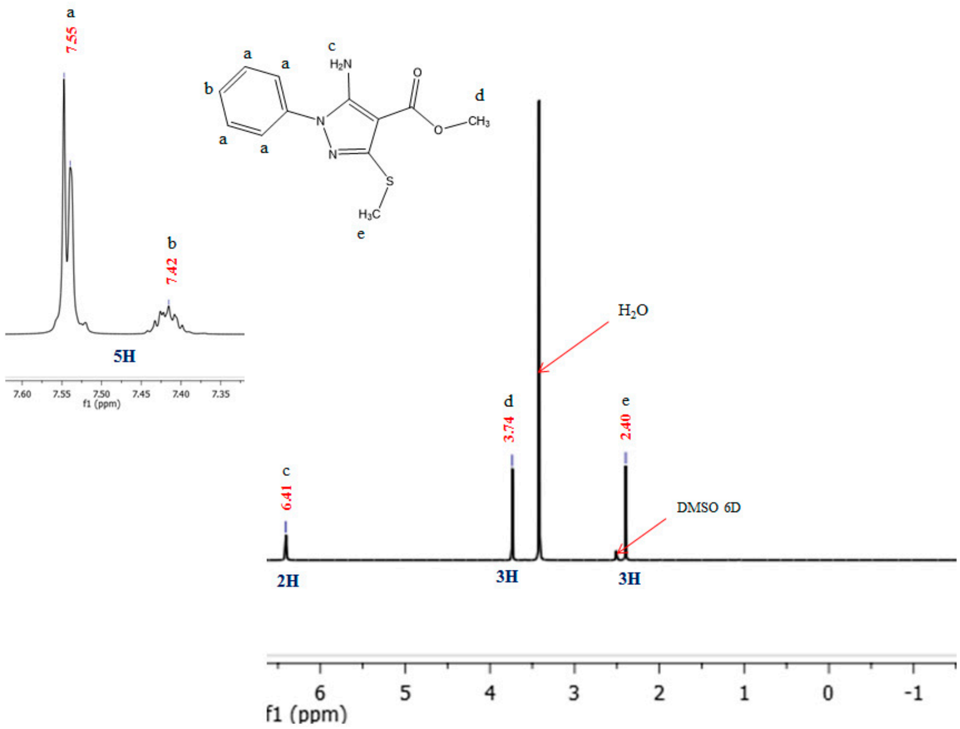

Figure S16. ^{1}H NMR spectrum (500 MHz, DMSO-d_6) of compound **13.**

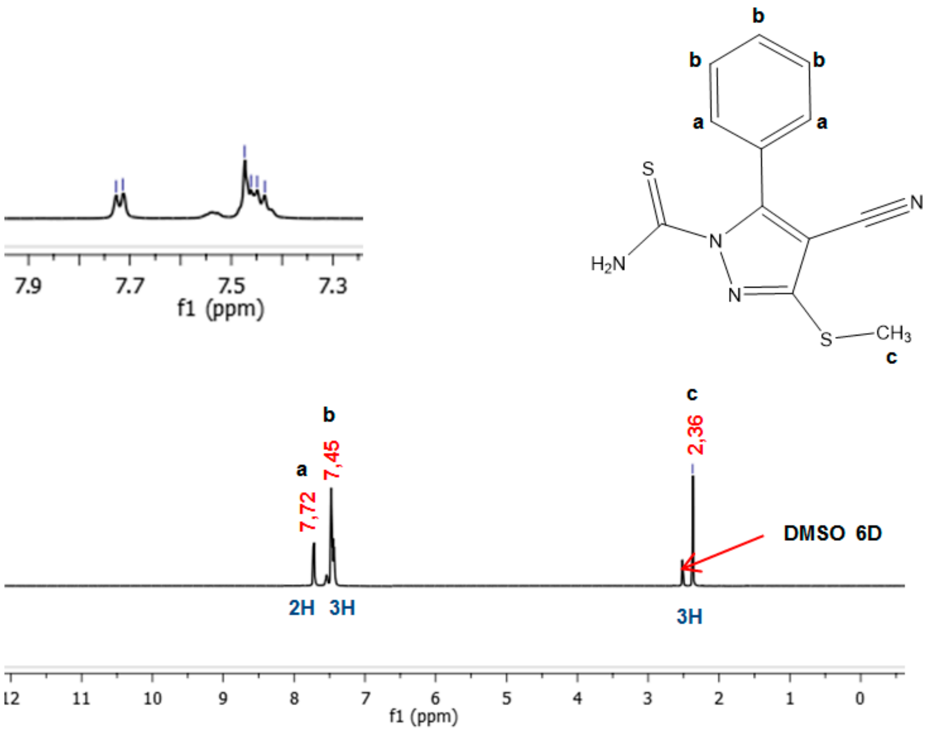

Figure S17. ^{1}H NMR spectrum (500 MHz, DMSO-d_6) of compound **14.**

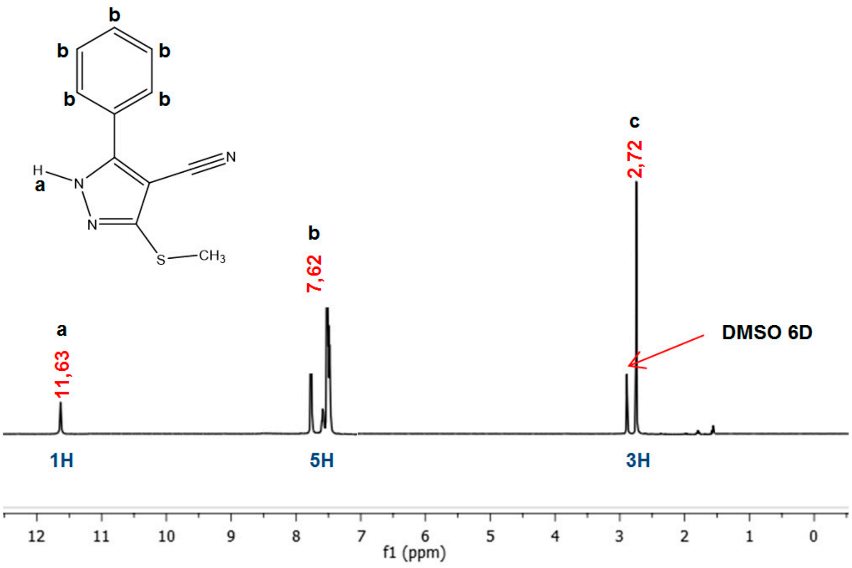

Figure S18. ^{1}H NMR spectrum (500 MHz, DMSO-d_6) of compound **15.**

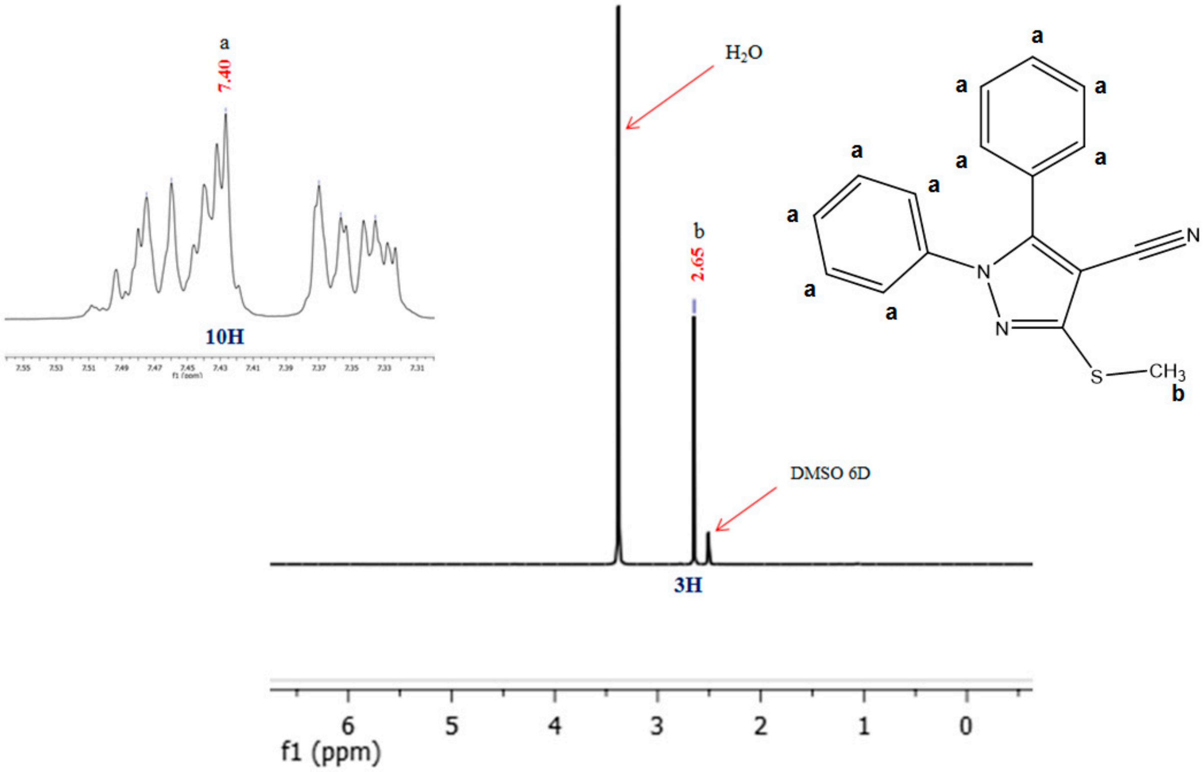

Figure S19. ^{1}H NMR spectrum (500 MHz, DMSO-d_6) of compound **16.**

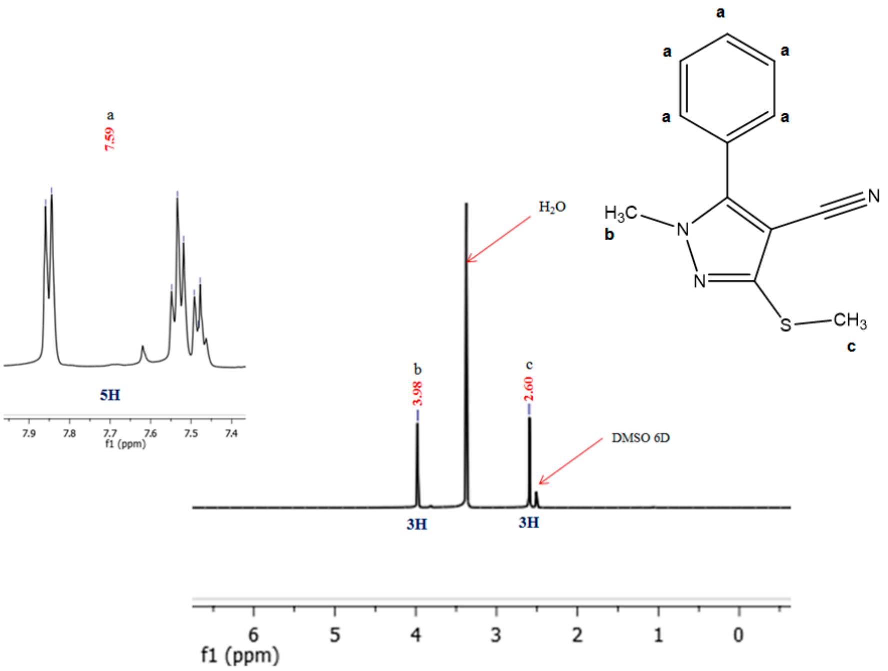

Figure S20. ^{1}H NMR spectrum (500 MHz, DMSO-*d*6) of compound **17.**

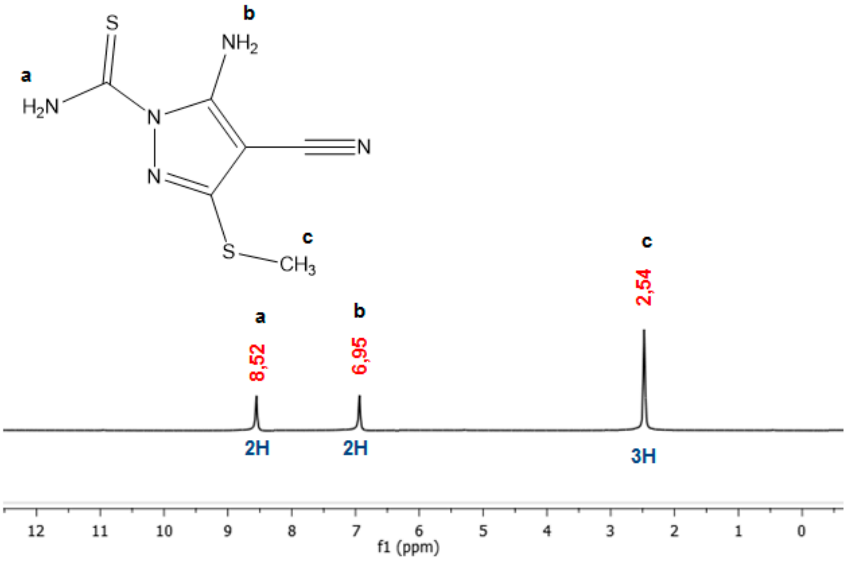

Figure S21. ^{1}H NMR spectrum (500 MHz, DMSO-*d*6) of compound **18.**

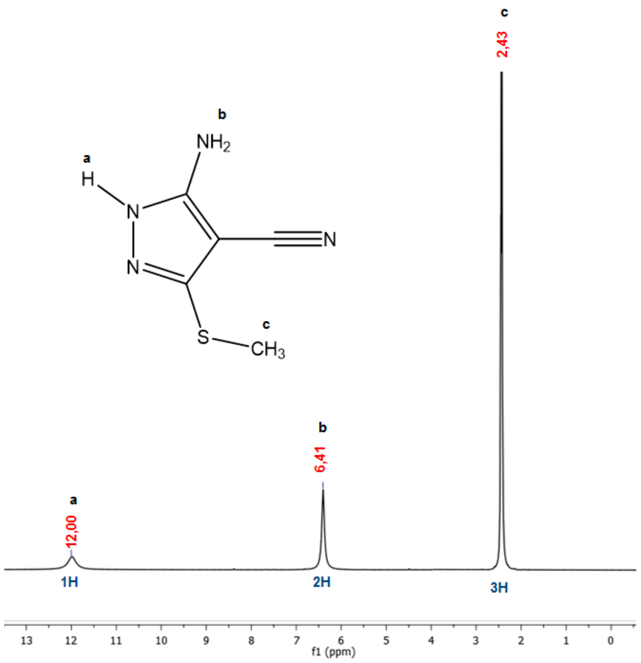

Figure S22. ^{1}H NMR spectrum (500 MHz, DMSO-d_6) of compound **19.**

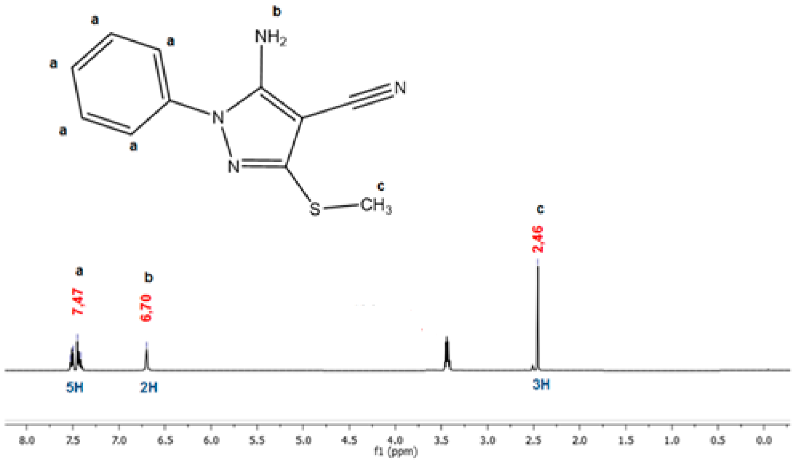

Figure S23. ^{1}H NMR spectrum (500 MHz, DMSO-d_6) of compound **20.**

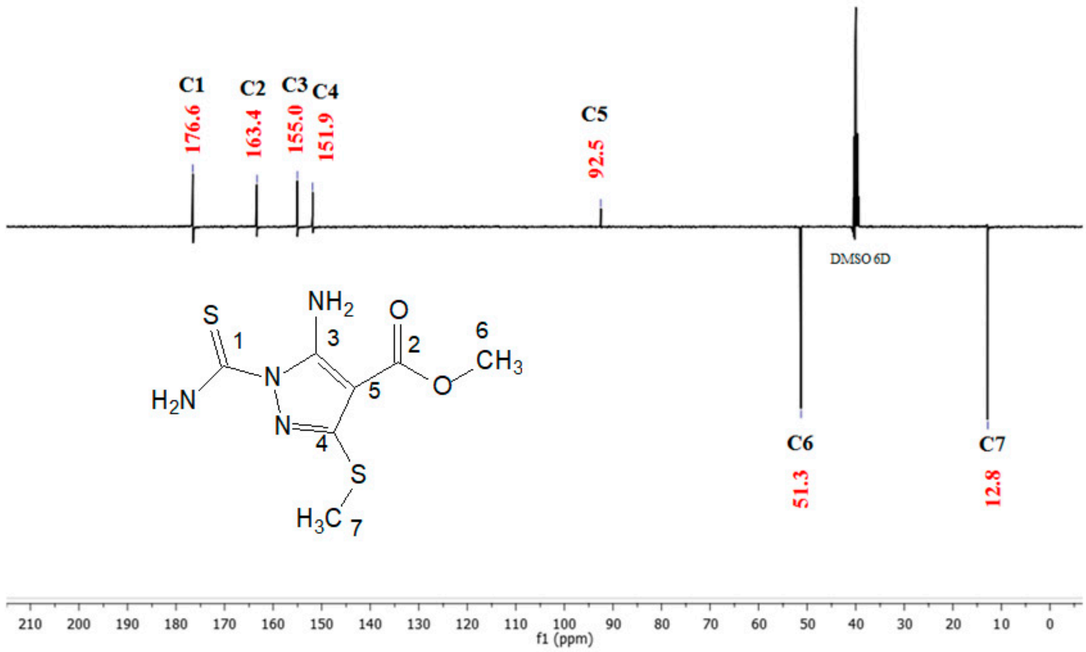

Figure S24. ^{13}C NMR spectrum (DEPQ, DMSO-*d*$_6$) of compound **11.**

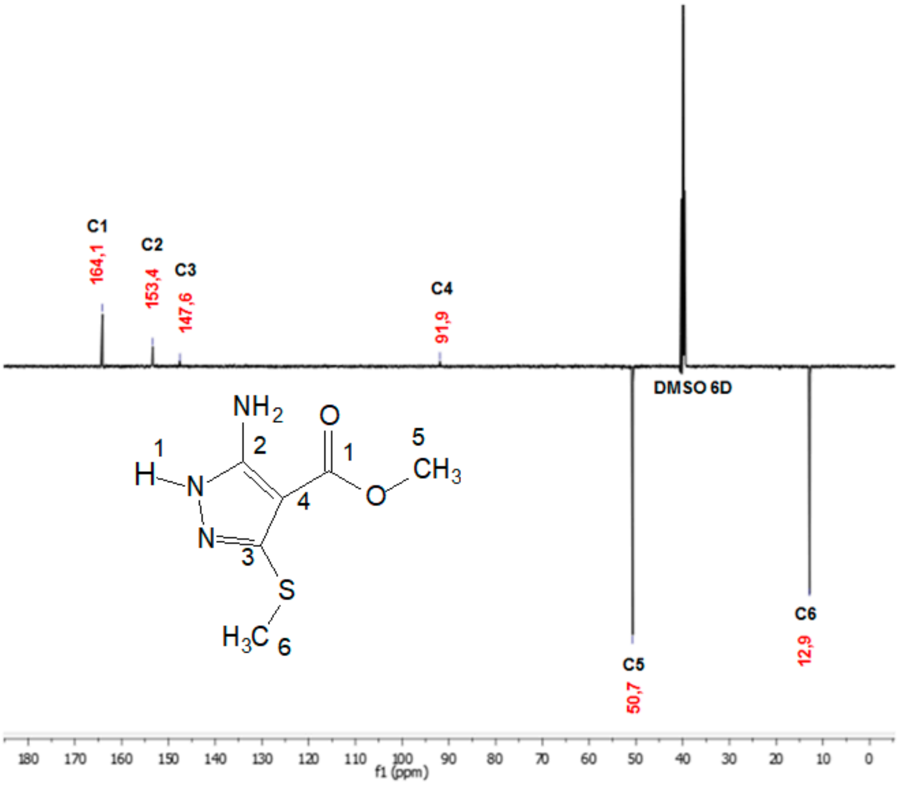

Figure S25. ^{13}C NMR spectrum (DEPQ, DMSO-*d*$_6$) of compound **12.**

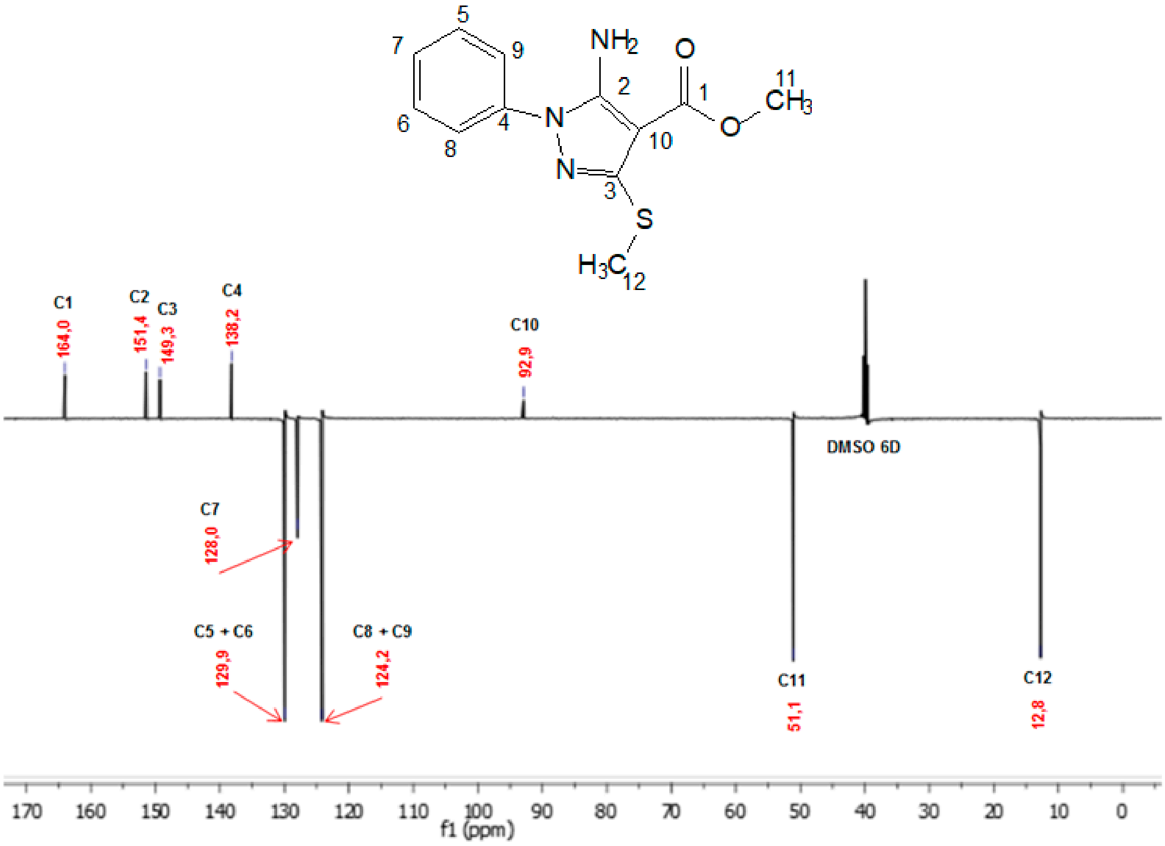

Figure S26. ^{13}C NMR spectrum (DEPQ, DMSO-d_6) of compound **13.**

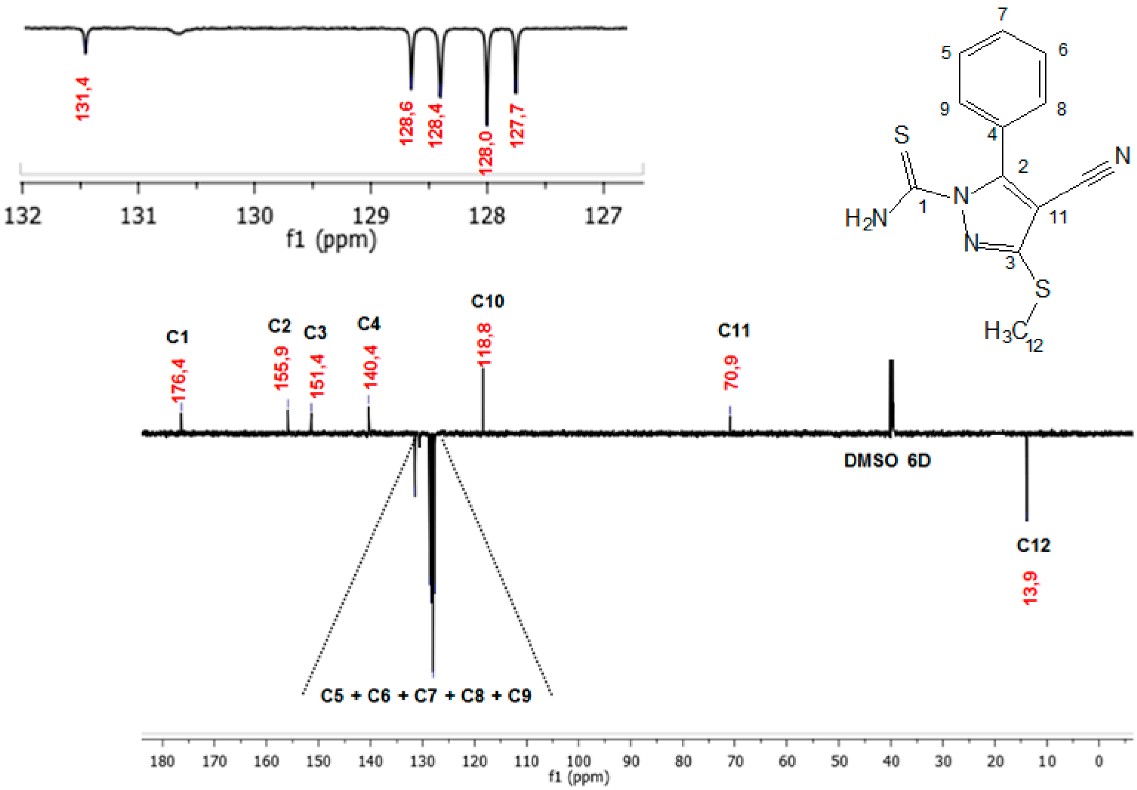

Figure S27. ^{13}C NMR spectrum (DEPQ, DMSO-*d*$_6$) of compound **14.**

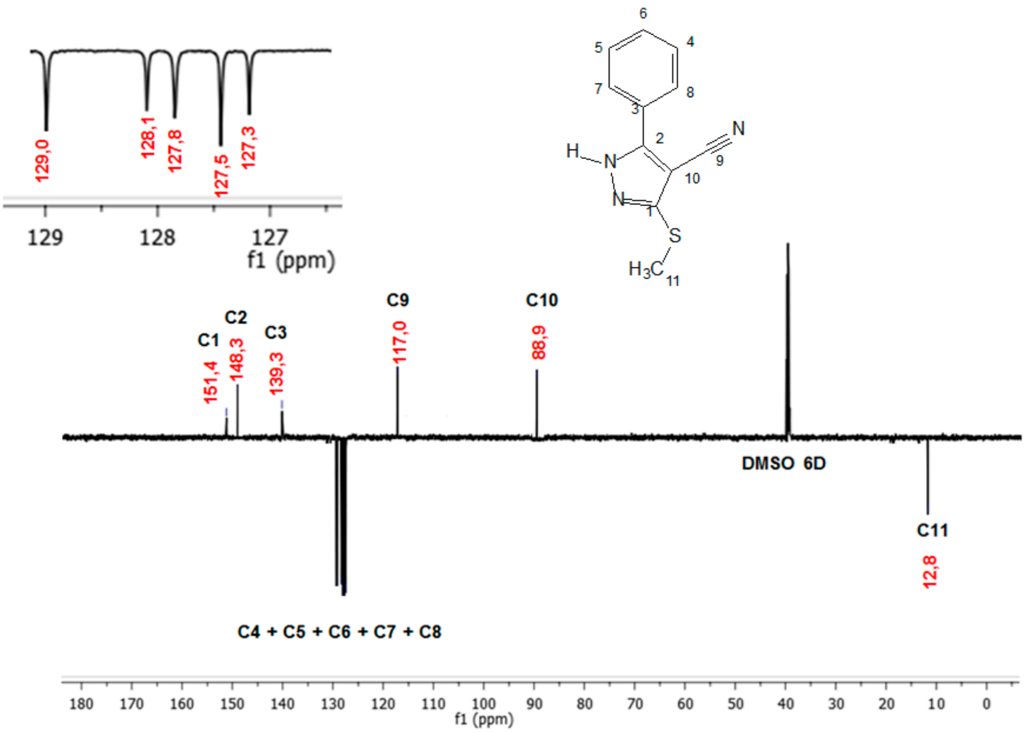

Figure S28. ^{13}C NMR spectrum (DEPQ, DMSO-*d*$_6$) of compound **15.**

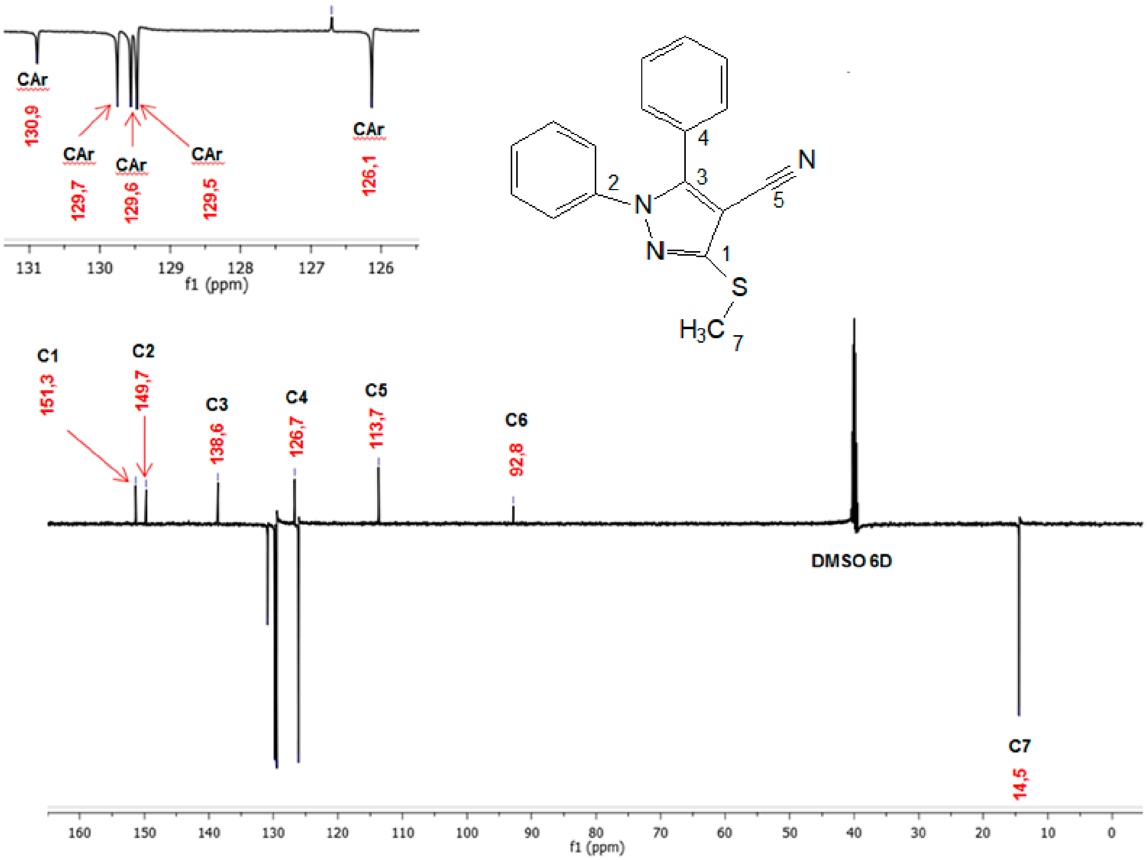

Figure S29. ^{13}C NMR spectrum (DEPQ, DMSO-*d*$_6$) of compound **16.**

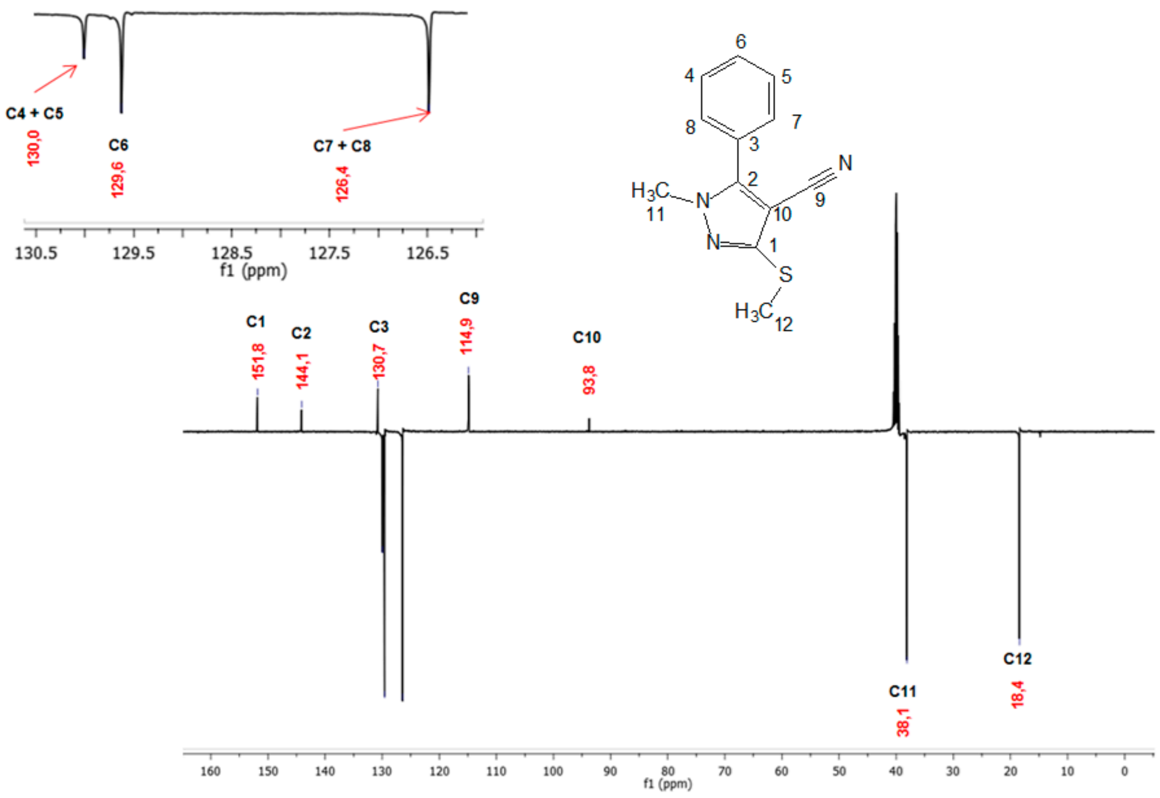

Figure S30. ^{13}C NMR spectrum (DEPQ, DMSO-d_6) of compound **17.**

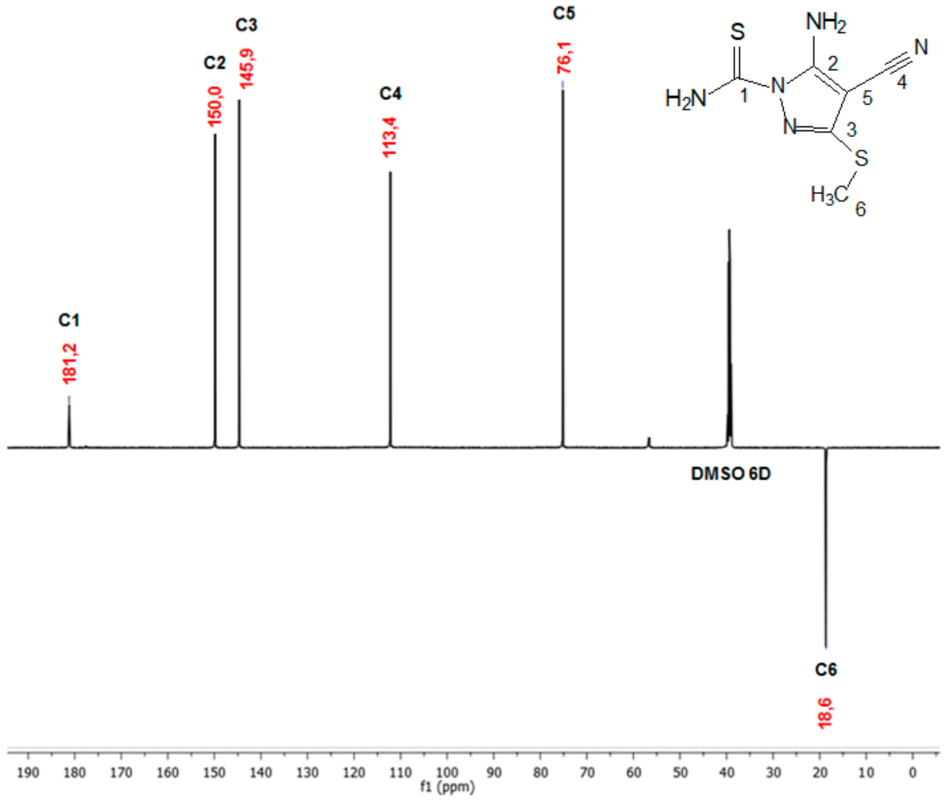

Figure S31. ^{13}C NMR spectrum (DEPQ, DMSO-d_6) of compound **18.**

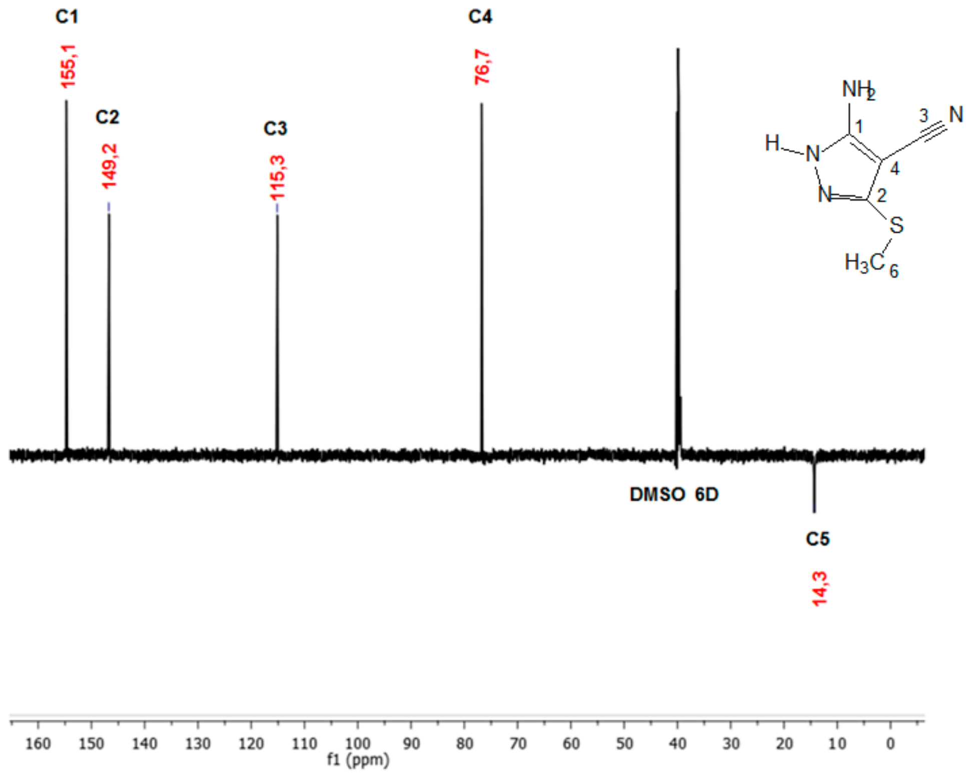

Figure S32. ^{13}C NMR spectrum (DEPQ, DMSO-d_6) of compound **19.**

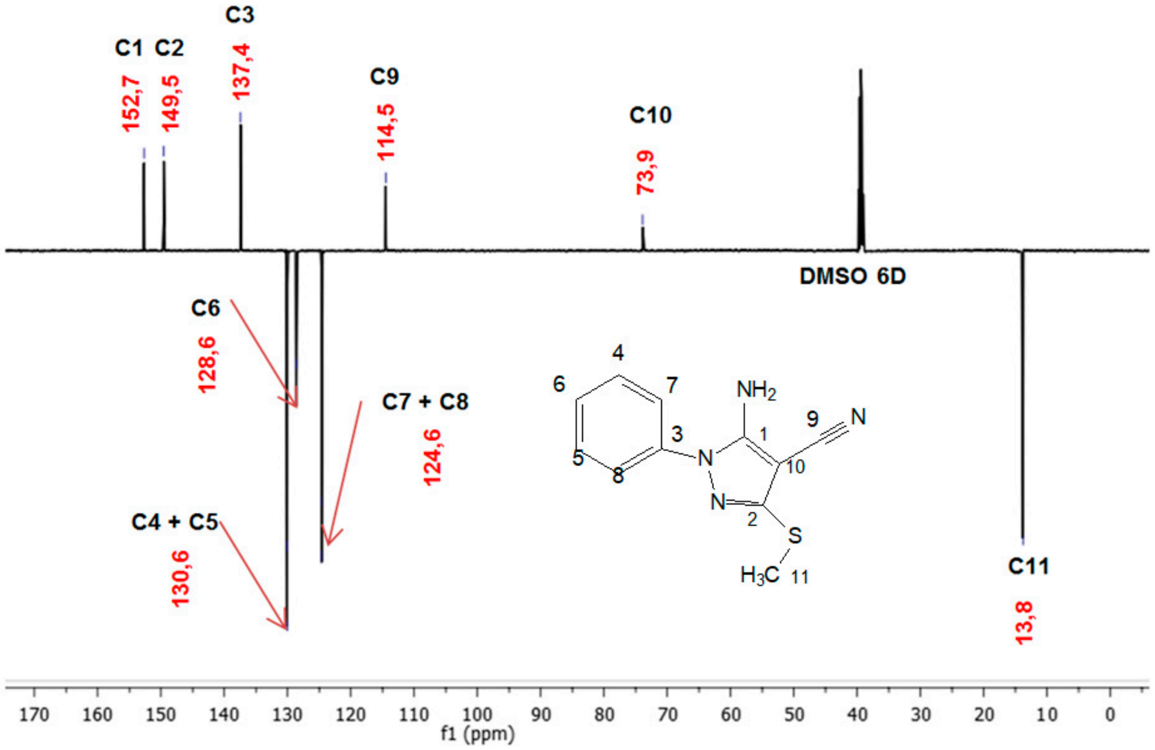

Figure S33. ^{13}C NMR spectrum (DEPQ, DMSO-d_6) of compound **20.**

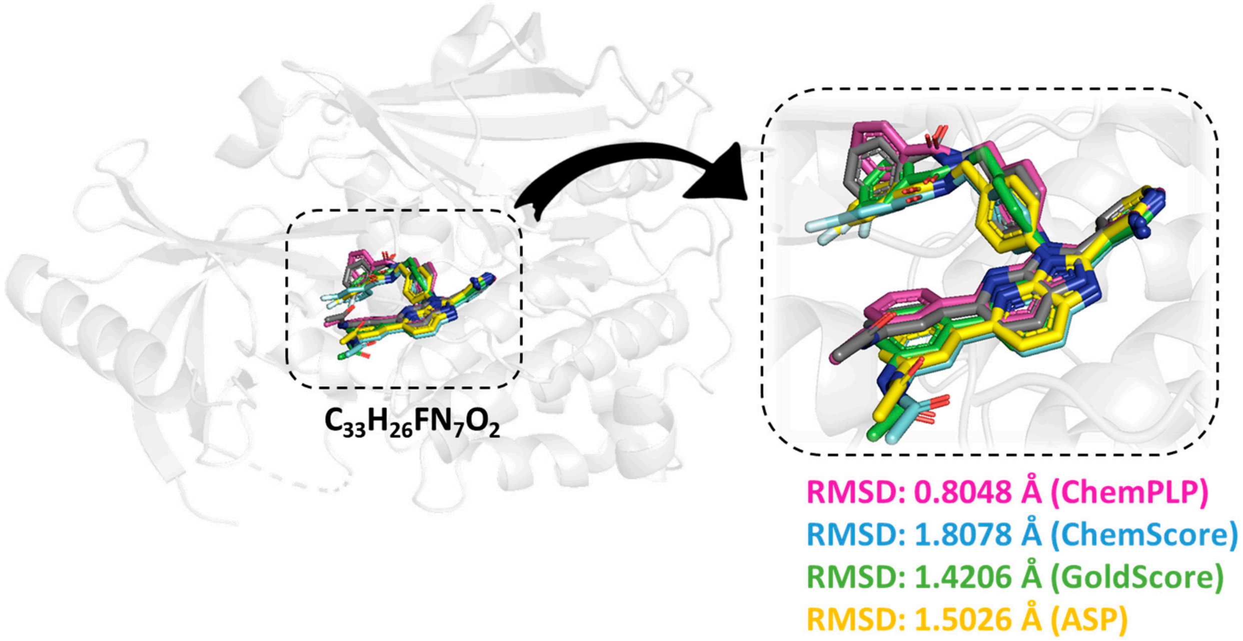

Figure S34. Superposition of the heterocyclic crystallographic AKT1 inhibitor *N*-(4-(5-(3-acetamidophenyl)-2-(2-aminopyridin-3-yl)-3*H*-imidazo[4,5-b]pyridin-3-yl)benzyl)-3-fluorobenzamide ($C_{33}H_{26}FN_7O_2$, PDB code 4EJN) and its corresponding best redocking pose using the molecular docking functions ChemPLP, ChemScore, GoldScore, and ASP. The root mean square deviation (RMSD) values for each docking function were highlighted with the corresponding color used in the stick representation. The crystallographically reported structure of $C_{33}H_{26}FN_7O_2$ is in stick representation in black. For better interpretation, hydrogen atoms were omitted.

Table S1. Physicochemical properties and lipophilicity obtained from the web server SwissADME.

Compound	log*P*	Number of H-acceptors	Number of H-donors	Number of Rotatable bonds	Molar refractivity	TPSA[1]
**11**	0.72	3	2	4	61.39	153.55
**12**	0.72	3	2	4	45.99	106.30
**13**	2.12	4	3	1	76.97	95.44
**14**	2.13	2	1	3	75.86	125.02
**15**	2.27	2	1	2	60.46	77.77
**16**	3.59	2	0	3	85.44	66.91
**17**	2.35	2	0	2	65.36	66.91
**18**	0.45	2	2	2	54.83	151.04
**19**	0.51	2	2	1	39.43	103.79
**20**	1.93	2	2	2	64.40	92.93

[1] TPSA: Topological Polar Surface Area.
